# Nuclease-free precise genome editing corrects MECP2 mutations associated with Rett syndrome

**DOI:** 10.3389/fgeed.2024.1346781

**Published:** 2024-03-01

**Authors:** Swati Bijlani, Ka Ming Pang, Lakshmi V. Bugga, Sampath Rangasamy, Vinodh Narayanan, Saswati Chatterjee

**Affiliations:** ^1^ Department of Surgery, Beckman Research Institute of the City of Hope, Duarte, CA, United States; ^2^ Center for Rare Childhood Disorders (C4RCD), Neurogenomics Division, Translational Genomics Research Institute (TGen), Phoenix, AZ, United States

**Keywords:** genome editing, Rett syndrome, MeCP2, adeno-associated virus, homologous recombination

## Abstract

Rett syndrome is an acquired progressive neurodevelopmental disorder caused by *de novo* mutations in the X-linked MECP2 gene which encodes a pleiotropic protein that functions as a global transcriptional regulator and a chromatin modifier. Rett syndrome predominantly affects heterozygous females while affected male hemizygotes rarely survive. Gene therapy of Rett syndrome has proven challenging due to a requirement for stringent regulation of expression with either over- or under-expression being toxic. Ectopic expression of MECP2 in conjunction with regulatory miRNA target sequences has achieved some success, but the durability of this approach remains unknown. Here we evaluated a nuclease-free homologous recombination (HR)-based genome editing strategy to correct mutations in the MECP2 gene. The stem cell-derived AAVHSCs have previously been shown to mediate seamless and precise HR-based genome editing. We tested the ability of HR-based genome editing to correct pathogenic mutations in Exons 3 and 4 of the MECP2 gene and restore the wild type sequence while preserving all native genomic regulatory elements associated with MECP2 expression, thus potentially addressing a significant issue in gene therapy for Rett syndrome. Moreover, since the mutations are edited directly at the level of the genome, the corrections are expected to be durable with progeny cells inheriting the edited gene. The AAVHSC MECP2 editing vector was designed to be fully homologous to the target MECP2 region and to insert a promoterless Venus reporter at the end of Exon 4. Evaluation of AAVHSC editing in a panel of Rett cell lines bearing mutations in Exons 3 and 4 demonstrated successful correction and rescue of expression of the edited MECP2 gene. Sequence analysis of edited Rett cells revealed successful and accurate correction of mutations in both Exons 3 and 4 and permitted mapping of HR crossover events. Successful correction was observed only when the mutations were flanked at both the 5′ and 3′ ends by crossover events, but not when both crossovers occurred either exclusively upstream or downstream of the mutation. Importantly, we concluded that pathogenic mutations were successfully corrected in every Rett line analyzed, demonstrating the therapeutic potential of HR-based genome editing.

## Introduction

Rett Syndrome is an acquired genetic neurodevelopmental disorder observed almost exclusively in females with an incidence of 1 in 10,000 female births worldwide (Hagberg, 1985; Amir et al., 1999; Petriti et al., 2023). It is caused by *de novo* mutations of the MECP2 gene located on chromosome Xq28 (Comings, 1986). Most Rett syndrome associated sporadic MECP2 mutations are of paternal origin and are transmitted to female progeny (Cheadle et al., 2000; Trappe et al., 2001; Good et al., 2021). They are thought to be a result of a combination of elevated levels of methylation and mitotic divisions in male germline cells (Driscoll and Migeon, 1990; Shahbazian and Zoghbi, 2002). Due to random X-chromosome inactivation (XCI), females with Rett Syndrome are mosaic for MeCP2 expression and display a wide spectrum of severity, while affected hemizygous males display more severe outcomes and rarely survive after birth (Neul et al., 2019). The onset of symptoms is observed after 6–18 months of age in affected females and is characterized by a progressive loss of cognitive language and motor skills, reduced brain growth, seizures, gait abnormalities, respiratory and digestive problems, repetitive hand and eye movements, anxiety and behavioral problems and intellectual disability (Shahbazian et al., 2002; Bellini et al., 2014; Ehrhart et al., 2016; Banerjee et al., 2019; Einspieler and Marschik, 2019).

The MECP2 gene encodes a 486 amino acid pleiotropic protein which functions as a methylation reader, chromatin modifier, transcriptional regulator and mRNA processor (Nan et al., 1997; Klose et al., 2005; Young et al., 2005; Long et al., 2011; Szulwach et al., 2011; Gonzales et al., 2012; Volkmann et al., 2013; Ausio, 2016; Good et al., 2021). It regulates the expression of thousands of genes in a DNA-methylation-dependent manner (Shah and Bird, 2017), mediating both activation and repression of transcription. The MeCP2 protein consists of 6 domains, the N-terminal domain (NTD), the methyl binding domain (MBD), interdomain (ID), transcription repressor binding domain (TRD) containing the NCoR/SMRT interaction domain (NID) and the C-terminal domain (CTD) (Ehrhart et al., 2016). MeCP2 is expressed in all tissues with the highest expression observed in brain (Shahbazian and Zoghbi, 2002) and is critical for neuronal function and development (Kishi and Macklis, 2004; Skene et al., 2010).

Rett Syndrome is a direct result of the loss of MeCP2 function. Over 300 mutations including missense, nonsense, frame shift, splice site, start codon mutations and deletions are located throughout the MECP2 gene and have been associated with Rett syndrome with different degrees of severity (Chahrour and Zoghbi, 2007; Shah and Bird, 2017; Sheikh et al., 2017; Martinez de Paz et al., 2019; Spiga et al., 2019; Good et al., 2021). Over 95% of mutations associated with Rett syndrome map to Exons 3 and 4 of the MECP2 gene (Krishnaraj et al., 2017). Majority of the Rett syndrome-associated missense mutations map to the MBD and span amino acids 78 to 162, including R106W, R133C, A140V, F155S, and T158M (Spiga et al., 2019). MBD mutations can affect the folding of MeCP2, influence binding to methylated nucleotides (Kucukkal et al., 2015; Yang et al., 2016), affect nuclear/cytoplasmic distribution and alter clustering of MeCP2 around pericentromeric heterochromatin (Good et al., 2021), change interactions between MeCP2 and other proteins including the chromatin modifier ATRX (Nan et al., 2007).

Several significant Rett syndrome-associated mutations are located in the CTD (Good et al., 2021) including those located between residues 295–486 affect the interaction of MeCP2 with chromatin (Nikitina et al., 2007; Chandler et al., 2021). Mutations in the CTD alter MeCP2-mediated organization of chromatin into chromocenters (Brero et al., 2005; Agarwal et al., 2011; Ausio, 2016; Wang et al., 2020) and disrupt the ability of MeCP2 to cluster heterochromatin (Li et al., 2020). C-terminal missense mutations including P302R, K304E, K305R, and R306C located within the TRD disrupt MeCP2 binding to the NCoR and alter recruitment of histone deacetylases (HDACs) (Kruusvee et al., 2017). Additionally, the RNA-binding motif of MeCP2 is located within the TRD (Castello et al., 2016), thus, C-terminal truncations of the MeCP2 protein likely have a global effect on splicing and gene expression.

Notably, overexpression of MeCP2, as observed in MECP2 duplication syndrome, also results in a severe neurodevelopmental disorder, demonstrating the critical importance of MECP2 gene dosage (Montgomery et al., 2018; Vashi and Justice, 2019; D'Mello, 2021). This requirement for stringent regulation of physiologic MeCP2 expression poses a significant obstacle for developing therapeutic strategies for Rett syndrome. The demonstration that symptoms associated with Rett syndrome could be reversed in mice by postnatal activation of silent MECP2 gene raised the possibility of gene therapy as a potential therapeutic option (Guy et al., 2007). Initial attempts to develop a gene therapy vector for Rett syndrome utilizing wild type or codon-optimized MECP2 under the control of either a heterologous or a minimal MECP2 promoter (Gadalla et al., 2013; Garg et al., 2013; Matagne et al., 2017), conferred modest improvement in lifespan and motor abilities in knockout mice, however, overexpression-related toxicity was encountered. The subsequent inclusion of portions of the MECP2 3′ untranslated region (UTR) containing miRNA binding sites in the transgene helped regulate MeCP2 expression in brain and reduce expression in the liver (Gadalla et al., 2017; Sinnett et al., 2017; Luoni et al., 2020), however, treated mice still exhibited deleterious behavioral effects possibly due to MeCP2 overexpression. Further modification of the MECP2 gene transfer vector to include a miniMECP2 transgene (Tillotson et al., 2017) and a combination of miRNA binding sites called ‘miRARE’ to regulate MeCP2 expression in the CNS (Sinnett et al., 2021) led to a reduction of toxicity, significantly improved survival and delayed the onset of severely abnormal gait in knockout mice. Two such MECP2 encoding AAV vectors are in early-stage clinical trials (NCT05606614, NCT05898620), one of which includes the miRARE sequence (TSH-102; NCT05606614). Despite recent progress in the regulation of MECP2 transgene expression, systemic physiologic expression level is needed in the CNS as well as peripheral organs (Shahbazian et al., 2002; Acampa and Guideri, 2006; Ehrhart et al., 2016; Kyle et al., 2016; Caffarelli et al., 2020; Sharifi and Yasui, 2021). In addition, the durability of MeCP2 transgene expression from episomal vectors remains unknown.

In contrast to gene transfer, which provides additional copies of the MECP2 transgene, genome editing directly corrects MECP2 mutations in the genome and retains all native regulatory elements including the full-length promoter and the entire 8.5 kb 3′UTR. Thus, genome editing of MECP2 mutations is likely to result in long-term systemic physiologic expression, and therefore, is expected to lead to better outcomes. Some success in this regard was achieved using CRISPR-mediated editing of the T158M and R270X mutations *in vitro* (Le et al., 2019; Croci et al., 2020), however, the use of nuclease-based editing strategy poses other significant concerns. Nuclease-based editing platforms involve creation of double-stranded breaks that are primarily repaired by non-homologous end-joining (NHEJ) pathway, thereby, resulting in insertion/deletion (indel) mutations or insertion of AAV ITRs at the target site (Bibikova et al., 2002; Maeder and Gersbach, 2016; Khirallah et al., 2023; Wang and Doudna, 2023). The use of nucleases also carries the additional risk of promiscuous off-target cutting, potentially resulting in genotoxicity (Fu et al., 2013; Pattanayak et al., 2013; Kuscu et al., 2014; Adikusuma et al., 2018; Kosicki et al., 2018; Fiumara et al., 2023; Tsuchida et al., 2023). In addition, the requirement for delivery of multiple editing elements to all target cells poses further challenges.

We reasoned that an editing platform that can efficiently revert MECP2 mutations to the wild-type sequence via high-fidelity homologous recombination (HR) without requiring exogenous nucleases, thus, avoiding the risk of collateral mutations would overcome significant challenges. The stem cell-derived adeno-associated viruses, AAVHSC, have previously been shown to mediate genome editing using the high-fidelity, precise BRCA2-dependent HR pathway in the absence of exogenous nucleases (Smith et al., 2018; Chen et al., 2020; Prout et al., 2023) and results in precise and seamless editing. Notably, AAVHSC vectors have been shown to cross the blood brain barrier and transduce the CNS broadly and uniformly (Ellsworth et al., 2019; Smith et al., 2022; St Martin et al., 2023). Thus, systemic delivery of AAVHSC-based MECP2 editing vector has the potential to permanently correct Rett associated MECP2 mutations and restore expression within the CNS and peripheral organs *in vivo*.

Here, we tested the hypothesis that genome editing using AAVHSC HR will precisely correct the pathogenic MECP2 mutations without inducing on-target mutations. We designed an AAVHSC genome editing vector to correct Rett-associated mutations located in Exons 3 and 4 of the MECP2 gene. The editing vector was also designed to insert a promoterless Venus open reading frame (ORF) immediately downstream of the coding region of Exon 4, to facilitate identification of edited cells. Here we show editing of the MECP2 gene in Rett patient-derived cells and demonstrate correction of MECP2 mutations at the sequence level. AAVHSC genome editing was found to be precise and seamless with no indel mutations or insertion of AAV ITRs. Notably, sequence analysis of edited genomes revealed that successful correction was only observed when the mutations were flanked at both the 5′ and 3′ ends by a crossover event. Importantly, restoration of MeCP2 expression was observed after AAVHSC editing in hemizygous male MECP2 mutant cells. Thus, AAVHSC HR offers a promising genome editing approach for the treatment of Rett syndrome.

## Methods

### Cell lines and reagents

Fibroblast cell line from the female Rett syndrome patient with R282X mutation was obtained from TGen. The remaining Rett patient-derived fibroblasts and EBV immortalized B-lymphoblastoid cells (B-LCLs) were obtained from the Coriell Institute (Table 1). All cell lines used in the study were de-identified. Cells were cultured as per the protocols recommended by Coriell Institute. Genomic DNA from each cell line was sequenced using Sanger sequencing to confirm the MECP2 mutation and to identify associated single nucleotide polymorphisms (SNPs).

**TABLE 1:** Editing of the MECP2 gene in Rett patient-derived cells with AAVHSC15-226 editing vector.

Rett cell line	Mutation	% Mutation frequency	Location	Domain	Cell type	Sex	% Editing
GM11273	R106W	2.79	Exon 3	MBD	Fibroblast	F	9.1
GM17538	S134C	0.44	Exon 4	MBD	B-LCL	M	3.8
GM17880	T158M	8.74	Exon 4	MBD	Fibroblast	F	8.6
R282X	R282X	5.74	Exon 4	TRD	Fibroblast	F	12.1
GM11270	R306C	5.14	Exon 4	TRD	Fibroblast	F	11.1

### Cloning and packaging of AAVHSC-226 editing vector

The MECP2 editing vector used was flanked at both the 5′ and 3′ ends by AAV2 ITRs. MECP2 genomic fragment from 550 bp upstream of Exon 3 to 800 bp of 3′ UTR downstream of coding region was amplified from HEK293 genomic DNA using primers SCO231 and SCO232. The amplicon was then cloned into the NdeI and NsiI sites of the AAV2 vector backbone. Linker 1 (L1) was inserted into the EcoN1 site in Intron 2 using overlapping primers SCO221 and SCO222 by restriction cloning. Linker 2 (L2) sequence was inserted into Intron 3 by Gibson cloning with overlapping primers AP1 MECP2 and AP2 MECP2. The sequences of primers used are listed in Supplementary Table S1.

The packaging and purification of AAVHSC editing vector was performed as previously described (Chatterjee and Wong, 1993; Fisher-Adams et al., 1996). Briefly, AAVHSC15 or AAVHSC7 RepCap and editing vector plasmids were transfected into HSV-1-infected HEK 293 cells. Recombinant single stranded AAVHSC vector was harvested from nuclei 72 h after transfection. Lysates were processed by freeze-thaw cycles, and sonication followed by extensive treatment with Benzonase. The vector was then purified through 2 rounds of CsCl_2_ gradient centrifugation. The vector titers were determined from a standard curve generated following qPCR with primers qVenus-Fwd, qVenus-Rev and Venus-probe. The primer sequences are listed in Supplementary Table S1.

### Transductions

The Rett fibroblasts were counted and seeded 24 h before transduction to allow adherence. B-LCLs were passaged 24 h before transduction and were seeded prior to transduction at required cell density. The primary human fibroblasts or B-LCLs were transduced at a confluency of 70%–90% in the recommended culture medium. Purified AAVHSC15- or AAVHSC7-226 editing vector was added at MOIs (multiplicity of infection) ranging from 150,000 to 450,000 vg (vector genomes)/cell, as indicated. Transduced cells were incubated at 37°C, 5% CO_2_ for 48 h before harvesting for further analysis.

### Flow cytometry and statistical analyses

Cells were harvested at 48 h post-transduction by trypsinization for fibroblasts and pipetting for B-LCLs. Cells were washed twice with PBS containing 2% FBS and resuspended in PBS containing 2% FBS. DAPI (Invitrogen, D3571) was used to determine the viability of cells at a final concentration of 3.3 µM. The cells were analyzed using Attune Nxt Flow Cytometer (Thermo Fisher Scientific). The major population of cells was first gated based on forward scatter and side scatter followed by gating of live cells by DAPI exclusion. Venus expression was then determined in live cell population. The data was analyzed using FlowJo software. Specific Venus expression in edited cells was quantified by subtracting the background as determined from untransduced cells.

Statistical analysis was performed using a two-sided, independent, two-sample *t*-test to compare editing efficiency between AAVHSC15-226 and AAVHSC7-226 for each cell type analyzed, per recommendations of the COH Biostatistics Core.

### Targeted integration (TI) assay

The cells were harvested 48 h after transduction and high-molecular weight genomic DNA was isolated using standard proteinase K digestion and phenol-chloroform purification procedures (Sambrook, 1989). TI assay was performed using a chromosome-specific primer located upstream or downstream of the homology arms and a primer specific to linker L2 in the insert. The sequences of primers are listed in Supplementary Table S1. Amplifications for 5′ TI were performed with Q5 high-fidelity polymerase (NEB). Cycling conditions used were: 3 min at 98°C; 15 cycles of 30 s at 98°C, 30 s at 72°C and decrease temperate by 0.5°C per cycle, 3 min at 72°C; 20 cycles of 30 s at 98°C, 30 s at 65°C, 3 min at 72°C; and 5 min at 72°C. Amplifications for 3′ TI were performed with KAPA HiFi HotStart polymerase (Roche). Cycling conditions used were: 5 min at 95°C; 15 cycles of 30 s at 95°C, 30 s at 72°C and decrease temperate by 0.5°C per cycle, 5 min at 72°C; 20 cycles of 30 s at 95°C, 30 s at 65°C, 3 min at 72°C; and 5 min at 72°C. Nested PCR was done from 3′ TI amplicon using a nested forward primer within L2 (Primer 4F) and nested reverse primer outside the 3′ homology arm (Primer 4R) using KAPA HiFi HotStart polymerase (Roche). The cycling conditions used were: 5 min at 95°C; 35 cycles of 30 s at 95°C, 30 s at 65°C, 3 min at 72°C; and 5 min at 72°C.

All the TI amplicons were cloned using the NEB PCR cloning kit (NEB, E1202S). Clones were sequenced by Sanger sequencing.

### Immunofluorescence

Cells were plated at 30,000 cells per well in 8-well Lab-Tek II chamber slide (Thermo Fisher Scientific) 24 h prior to transduction. 24 h after plating, cells were transduced with AAVHSC7-226 at a MOI: 150,000. Before staining, cells were washed with PBS and fixed with 4% paraformaldehyde 48 h after transduction. They were then rinsed 3-times with PBS and blocked with PBS containing 3% BSA and 0.3% Triton X-100 for 1 h at room temperature. This was followed by incubation with primary anti-MeCP2 antibody (1: 100 dilution; Cell Signaling, #3456) and an anti-GFP antibody (1:500 dilution, Thermo Fisher, # MA5-15256) in PBS containing 1% BSA and 0.3% Triton X-100 at 4°C overnight. The cells were then rinsed 3-times with PBS and incubated with secondary antibodies. Secondary antibodies used were anti-rabbit Alexa Fluor-555 IgG (1:500, cell Signaling, #4413) and anti-mouse Alexa Fluor-488 IgG (1:500, cell Signaling, #4408) in PBS containing 1% BSA and 0.3% Triton X-100 for 2 h at room temperature in dark. The cells were finally rinsed 3-times with PBS, mounted with antifade reagent containing DAPI (VECTASHIELD Vibrance^®^, #H-1800) and visualized at ×40 magnification using the Zeiss LSM 700 confocal microscope.

### RNA purification and quantitative RT-PCR (qRT-PCR)

The untransduced and AAVHSC7-226 transduced fibroblasts (r.378_384del) were harvested 48 h after transduction. RNA was purified from harvested cells using NucleoSpin RNA Plus XS kit (Macherey-Nagel) as per the manufacturer’s instructions. An additional on-column DNase digestion was performed using RNase-free DNase (Qiagen, # 79256), while purifying RNA. The RNA was then used as a template to synthesize cDNA using the SuperScript™ IV First Strand Synthesis kit using an oligo (dT) primer (Invitrogen, #18091050). The resultant cDNA was amplified by qPCR using primers Ex3-4-Fwd-qPCR and Ex4-Rev-qPCR (Supplementary Table S1). COX4I1 (Taqman Gene Expression Assay Hs00971639_m1) served as a reference gene to measure the relative MECP2 transcript expression in unedited and edited cells. Fold change in MECP transcript expression post editing was calculated using unedited cells as baseline.

## Results

### HR-based genome editing strategy to correct MECP2 mutations

**FIGURE 1:**
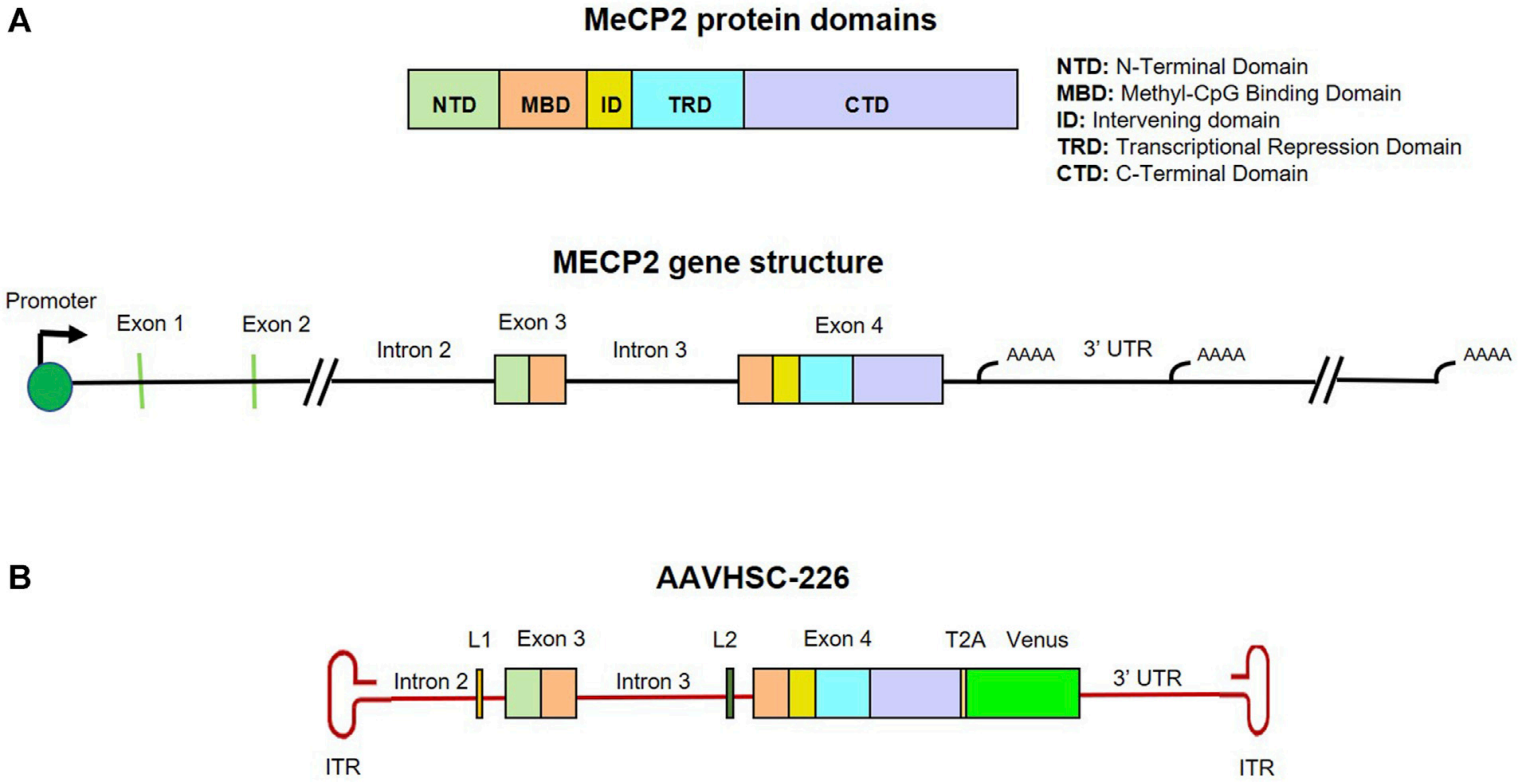
Map and structure of MECP2 gene, protein and editing vector. **(A)** Genomic structure and protein domains of MECP2. MeCP2 protein domains and their corresponding locations in Exon 3 and 4 are color coded. **(B)** Map of the single-stranded AAVHSC-226 editing vector genome. The editing vector was designed to correct mutations in MECP2 Exons 3 and 4 and two unique linker sequences L1 and L2 were included in Introns 2 and 3, respectively. The editing vector was also designed to insert a promoterless T2A-Venus ORF cassette immediately downstream of Exon 4.

We designed a nuclease-free HR-based genome editing strategy to correct pathogenic Rett syndrome-associated MECP2 mutations located in Exons 3 and 4 using the AAVHSC editing platform (Smith et al., 2018; Chen et al., 2020; Prout et al., 2023). The editing vector consisted of an editing moiety that was 100% homologous to the corresponding region of the wild type human MECP2 gene (NCBI genome assembly version GRCh38.p14). This editing moiety consisted of 550 bp of Intron 2, all of Exon 3, Intron 3, Exon 4 and 800 bp of 3′ UTR (Figure 1A). The resultant genome editing vector termed AAVHSC-226 editing vector, consisted of a wild type 3,570 bp editing element which was flanked by AAV2 ITRs. The editing vector was designed to replace the corresponding region in the mutant genome with the wild type editing moiety from the editing vector following HR mediated 5′ and 3′ crossovers. To facilitate identification of edited cells, the vector additionally encoded a promoterless Venus ORF immediately downstream of Exon 4 and upstream of the 3′ UTR (Figure 1B). Successful HR would result in the targeted insertion of the promoterless Venus ORF into the genome at the end of the coding region. The Venus ORF was preceded by a T2A self-cleaving peptide sequence to allow independent expression of Venus driven by chromosomal MECP2 promoter and utilized the natural MECP2 polyadenylation and transcriptional termination sites. Notably, in our editing strategy, the entire chromosomal MECP2 promoter and all native regulatory elements including all naturally occurring miRNA binding sites are preserved in the edited genome which should preserve full physiologic regulation of edited MECP2 expression. The Venus reporter was inserted to reflect physiologic MeCP2 expression and served as a readout for successful in-frame targeted editing of the MECP2 gene.

The AAVHSC-226 editing vector also included 2 linkers, the 28 bp long L1 and the 33 bp long L2 located within Introns 2 and 3, respectively (Figure 1B). The linkers were included to serve as unique primer binding sites for subsequent analysis of edited genomes. The MECP2 editing vectors were packaged in either AAVHSC7 or AAVHSC15 and evaluated for editing of the MECP2 gene in Rett syndrome patient-derived cells.

### Genome editing of the MECP2 gene in Rett syndrome cells

We evaluated editing of the MECP2 gene in cells derived from 5 different Rett syndrome patients, bearing the R106W, S134C, T158M, R282X and R306C mutations. Venus expression in edited cells was driven by the chromosomal MECP2 promoter and served as a surrogate marker for editing. Editing efficiency was evaluated based on flow cytometric evaluation of Venus expression 48 h after transduction with AAVHSC15-226 at a MOI: 150,000. The overall editing frequency, as determined from Venus expression ranged from 3.8% to 12.1% (Table 1), with 8.6%–12.1% editing observed in fibroblasts and 3.8% in B-LCLs (Table 1). Whether the lower frequency of editing observed in B-LCLs as compared with fibroblasts reflected lower transduction efficiencies or lower editing efficiencies is unclear. Together these data indicated the feasibility of editing the MECP2 gene in Rett patient-derived cells using AAVHSC15-226.

#### Genome editing of the MECP2 gene is dose dependent

**FIGURE 2:**
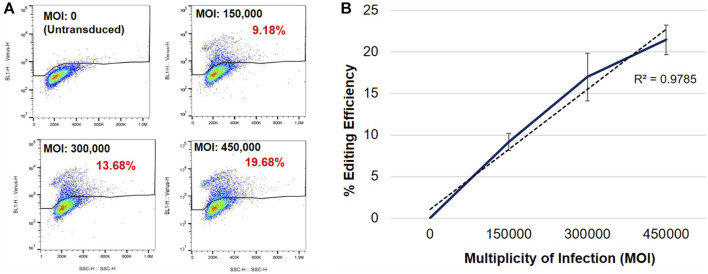
Specific Venus expression in edited R282X Rett syndrome fibroblasts. **(A)** Flow cytometric analysis of Venus expression in R282X fibroblasts 48 h post-transduction. Cells were transduced with AAVHSC15-226 at MOIs: 0, 150,000, 300,000 and 450,000. **(B)** Editing efficiency as a function of the MOI. Graph representing average editing efficiency in R282X cells at increasing vector dose (n = 3). Editing efficiency was calculated from specific Venus expression in transduced R282X fibroblasts.

To determine whether genome editing efficiency was proportional to the vector dose, we evaluated editing efficiency at increasing MOIs in female heterozygous R282X fibroblasts (Figure 2). Flow cytometric analyses of transduced cells revealed that the frequency of Venus expression increased with increasing MOI (Figures 2A, B). Mean MECP2 editing efficiencies of 9.17%, 16.99%, and 21.46% were observed at MOIs 150,000, 300,000 and 450,000, respectively (Figure 2B), indicating that editing efficiency was directly proportional to the vector dose.

#### Comparison of MECP2 editing by AAVHSC7 and AAVHSC15

**FIGURE 3:**
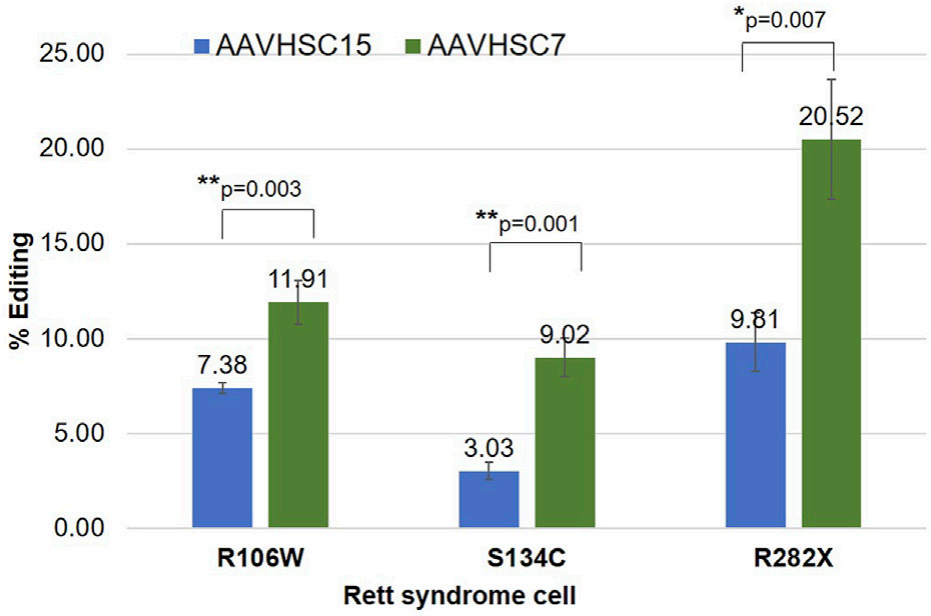
Comparison of MECP2 editing by AAVHSC15 and AAVHSC7 in Rett syndrome cells. Heterozygous female R106W Rett syndrome fibroblasts (GM11273), hemizygous male S134C B-LCLs (GM17538) and heterozygous female R282X fibroblasts were transduced with either AAVHSC15-226 or AAVHSC7-226 editing vectors at MOI:150,000 (n = 3). Editing efficiency was determined by specific Venus expression 48 h post-transduction. Statistical analysis was performed using a two-sided, independent, two-sample *t*-test and the significance is shown (*p < 0.05; **p < 0.005).

We next asked if the AAVHSC serotype affected the editing efficiency of the MECP2 gene. AAVHSC-226 was independently packaged in either AAVHSC7 or AAVHSC15 capsid and their editing efficiency compared. The choice of these 2 capsids was based on previous studies which showed that AAVHSC7 editing vectors mediate HR in human cells at higher efficiencies, while AAVHSC15 is efficient *in vivo* in mice (Smith et al., 2018). Heterozygous female fibroblasts bearing either the R282X or the R106W mutation and male hemizygous B-LCLs with the S134C mutation were transduced with either AAVHSC7- or AAVHSC15-226 editing vector at MOI:150,000. Flow cytometric analysis of Venus expression at 48 h post-transduction showed that while both AAVHSC serotypes edited the MECP2 gene in each of the 3 cell lines, editing with AAVHSC7 was more efficient on fibroblasts and B-LCL *in vitro* (Figure 3). Together these results confirmed our previous findings that editing efficiencies correlated with AAV serotypes and demonstrated that successful editing of the MECP2 gene by AAVHSC editing vectors in Rett syndrome patient-derived cells.

### Sequence confirmation of MECP2 editing in Rett syndrome cells

**FIGURE 4:**
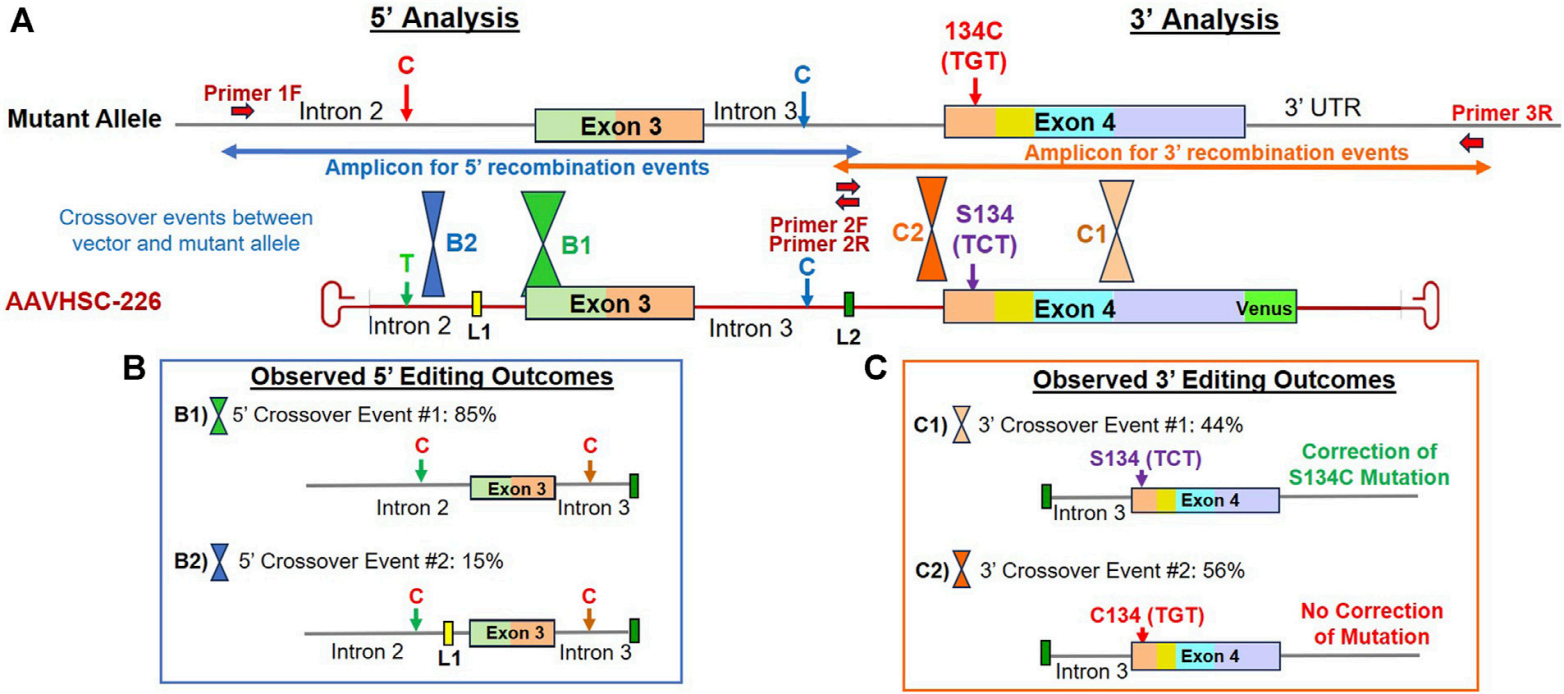
Sequence analysis of the edited MECP2 gene in hemizygous male S134C Rett syndrome cells. **(A)** Editing of the MECP2 gene in S134C cells. Shown are the mutant S134C genome and the AAVHSC-226 editing vector. Also shown are: i) positions of SNPs which differed between the editing vector and the S134C genome, ii) location of linkers L1 and L2, iii) forward and reverse primers, iv) the S134C mutation. The primers used for TI analyses are depicted as thick red arrows. Primers 1F and 3R are specific for chromosomal sequences external to the homology arm. Primer 2R is specific for linker L2. Positions for HR crossover events between the mutant genome and the editing vector genome were identified by the presence or absence of markers in the edited genome and are depicted. **(B)** Observed 5′ editing outcomes. Two editing outcomes identified at the 5′ end based on the SNPs and linker sequences as markers are shown. **(C)** Observed 3′ editing outcomes. Two editing outcomes identified at the 3′ end based on the SNPs and linker sequences as markers are shown.

To confirm editing of the MECP2 gene at the sequence level, we analyzed the edited region using TI assays in two overlapping sections. The 5′ and 3′ portions of the edited MECP2 region were analyzed by contiguous TI assays providing coverage of the entire edited region which extended from within Intron 2 to the 3′ UTR and included chromosomal sequences external to the homology arms. For every TI assay, amplifications were performed using one chromosome-specific primer, either 1F or 3R (Figure 4), which annealed to genomic sequences external to the region of homology between the editing vector and the target MECP2 region. The second primer was complementary to linker L2 located in Intron 3. This amplification strategy ensured that only edited genomes containing L2 were analyzed and that vector genomes were not inadvertently included in the analyses (Smith et al., 2018). Editing of the MECP2 gene was analyzed in hemizygous male B-LCLs with S134C mutation and heterozygous female fibroblasts with R106W and R282X mutations.

### Sequence analyses of genome editing outcomes of the S134C mutation in hemizygous male Rett cells

We first analyzed editing of the MECP2 gene in hemizygous male B-LCLs bearing the S134C mutation in Exon 4 of the MECP2 gene. Male cells contain a single mutant X chromosome with a C:G mutation at nucleotide (nt) 401 of the MECP2 coding sequence. Thus, successful editing of this allele should revert the phenotype from mutant to wild type. Since HR requires crossovers between the editing vector and the template genome within the region of homology, we attempted to localize the crossover events using 4 sets of markers. These included i) the presence or absence of a SNP located in Intron 2, ii) the insertion of L1 in Intron 2, iii) insertion of L2 in Intron 3, iv) the presence of either the wild type C or the mutant G at nt 401 in Exon 4 encoding the S134C mutation (Figure 4A).

#### 5′ editing outcomes

The 5′ editing outcomes were analyzed by the TI assay using a chromosome-specific forward primer, Primer 1F (Figure 4A) which annealed to Intron 2 genomic sequence external to the region of homology and a reverse primer, Primer 2R, specific for linker L2 (Figure 4A; Supplementary Figure S1A). The SNP in Intron 2 consisted of a T on the AAVHSC-226 editing vector and a C at the same position in the S134C genome (Figure 4A). Sequence analysis indicated that all clones analyzed contained a C at the SNP in Intron 2, indicating that all observed 5′ HR crossover events occurred downstream of this SNP. Sequence analyses indicated 2 editing outcomes at the 5′ end (Figure 4B; Supplementary Figure S1B).

**Editing outcome B1** accounted for 85% of the 5′ HR events analyzed, contained L2 in Intron 3, however, L1 was absent from Intron 2 (Figure 4B; Supplementary Figure S1B; Supplementary Sequence S1: BankIt accession number OR858751). This suggested that the 5′ crossover event had occurred downstream of L1 but upstream of L2.

**Editing outcome B2** represented 15% of the 5′ HR events analyzed (Figure 4B; Supplementary Figure S1B; Supplementary Sequence S2: BankIt accession number OR858752). It contained both linkers L1 and L2, indicating that the 5′ crossover event occurred upstream of L1 but downstream of the C/T SNP in Intron 2.

#### 3′ editing outcomes

The 3′ editing outcomes were analyzed using Primer 2F, specific for L2 (Figure 4A) located within Intron 3 and the chromosome-specific reverse primer 3R specific for 3′ UTR sequence downstream of the region of homology on the editing vector (Figure 4A; Supplementary Figure S2A). The resulting TI amplicon spanned part of Intron 3, all of Exon 4, and part of the 3′ UTR (Figure 4A). Two editing outcomes were identified. In this analysis, the T2A-Venus sequence containing TI amplicons were excluded as they were larger in size and did not amplify efficiently. The absence of the Venus cassette in the amplicon analyzed indicated that the 3′ crossover resulting in the observed 3′ editing outcomes occurred upstream of the Venus cassette.

**Editing outcome C1** Sequence analysis indicated that the S134C mutation was corrected in 44% of recombination events analyzed, representing Editing Outcome C1 (Figure 4C; Supplementary Figure S2B; Supplementary Sequence S3: BankIt accession number OR858753). The resulting edited genome included linker L2 in Intron 3 and had the wild type C at nt 401 in Exon 4. This confirmed that editing resulted in the correction of the S134C mutation where the mutant codon TGT encoding cysteine was edited to the wild type codon, TCT encoding serine. Thus, AAVHSC editing restored the wild type sequence of the MECP2 gene in GM17538 cells, successfully correcting the S134C mutation.

**Editing Outcome C2** accounted for 56% of 3′ recombination events analyzed. The presence of linker L2 in Intron 3 indicated successful editing. However, the C:G mutation at nt 401 of Exon 4 was not corrected, resulting in retention of the S134C mutation in the edited cells (Figure 4C; Supplementary Figure S2B; Supplementary Sequence S4: BankIt accession number OR858754). These results indicated that the 3′ crossover event was located upstream of S134C, resulting in the retention of the mutant nucleotide G.

We conclude that due to sequence identity between the editing vector and the genomic MECP2 sequence, HR-based 5′ and 3′ crossover events may be located anywhere within the region of homology. Successful correction of the MECP2 mutation depended upon the location of the crossover events, as was the genomic insertion of the T2A-Venus ORF. As a result, correction of the S134C mutation was observed in 44% instead of 100% of all recombination events despite successful editing.

### Sequence analysis of genome editing outcomes observed in heterozygous R106W female cells

**FIGURE 5:**
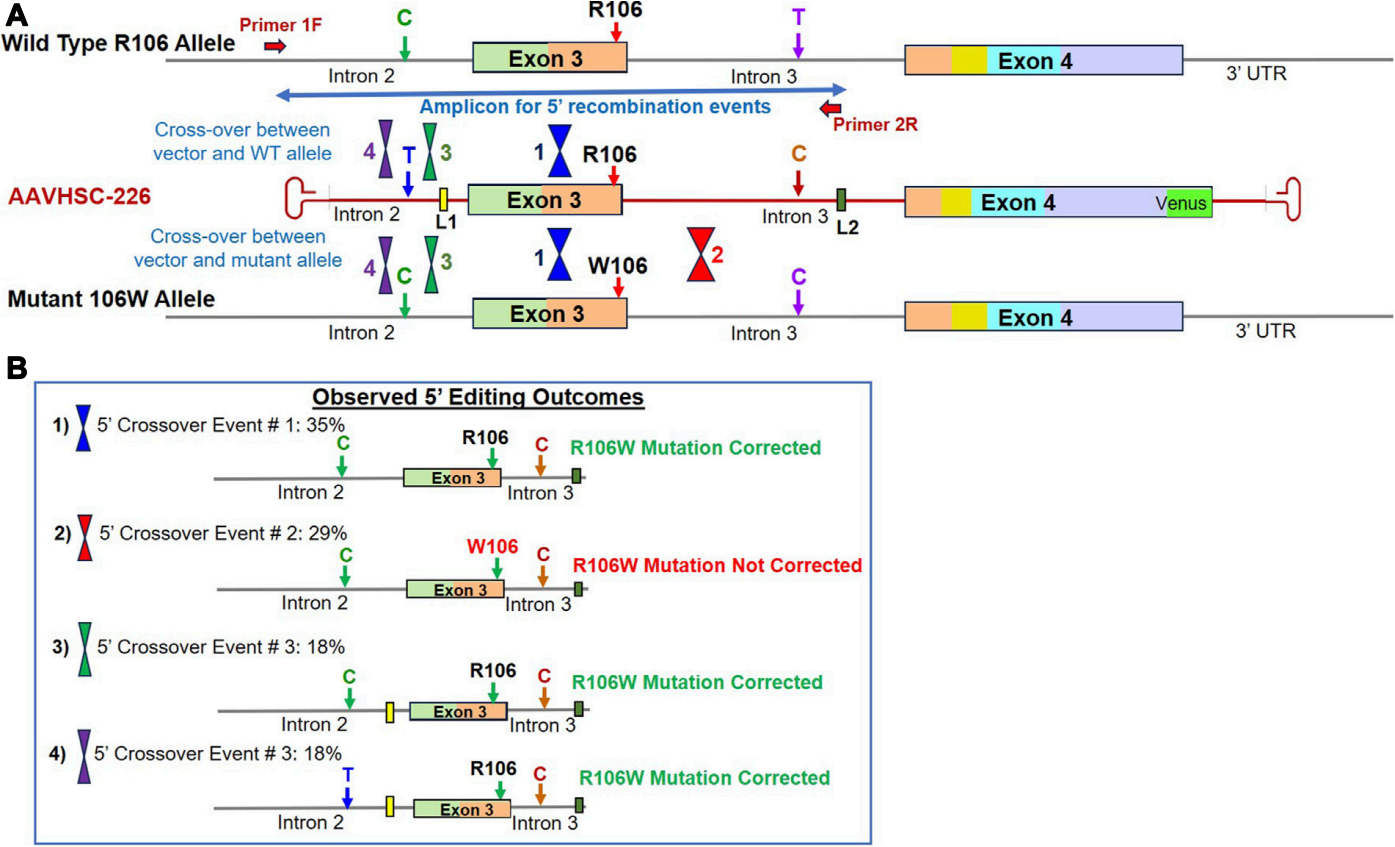
Sequence analysis of the edited MECP2 gene in heterozygous female R106W Rett syndrome cells. **(A)** Genome editing of the MECP2 gene in R106W cells. Shown are the WT R106 allele, AAVHSC-226 editing vector and the mutant 106W allele. Also shown are the locations of HR crossover events between the editing vector genome and either the WT R106 genome or the mutant 106W genome. The primers used for TI analyses are depicted as thick red arrows. Primer 1F is specific for chromosomal sequences external to the homology arms. Primer 2R is specific for linker L2. The crossover positions identified based on the presence or absence of markers in the edited genome and are depicted. **(B)** Observed editing outcomes. Four editing outcomes identified at the 5′ end based on the SNPs and linker sequences as markers are shown.

We next investigated correction of pathogenic MECP2 mutations in heterozygous female Rett cells bearing the R106W mutation located in Exon 3. In heterozygous female cells which possess two X chromosomes, editing could either be monoallelic or biallelic. And the editing vector could recombine via HR with either the wild type or the mutant allele. As described above, 5′ portion of edited genomes was amplified using the chromosome-specific primer, 1F, external to vector-encoded homology arms and another primer specific for linker L2 (Supplementary Figure S3A). Locations of HR-related crossovers were mapped using 3 genomic markers. These included i) a C/T SNP in Intron 2, ii) a C SNP in Intron 3 of both the editing vector and the W106 mutant allele, while the WT R106 allele encoded a T SNP at this position, and iii) insertion of vector-encoded linkers, L1 and L2, in Introns 2 and 3, respectively (Figure 5A). Sequence analysis of the 5′ portion revealed 4 distinct patterns of recombination in edited R106W cells (Figure 5B; Supplementary Figure S3B).

**Editing outcome 1** represented 35% of recombination events and was characterized by the presence of the genomic C SNP, absence of L1 in Intron 2, presence of the wild type R106 codon in Exon 3, C SNP and L2 in Intron 3 (Figure 5B; Supplementary Figure S3B; Supplementary Sequence S5: BankIt accession number OR858755). This editing outcome could have been generated by either of 2 possible crossover events: i) editing of the wild type allele with the 5′ crossover event located downstream of Linker L1 but upstream of the SNP in Intron 3, or ii) editing of the mutant allele with the 5′ crossover event located upstream of W106, resulting in correction of mutation along with insertion of L2.

**Editing outcome 2** represented 29% of recombination events and was characterized by the retention of the R106W mutation (Figure 5B; Supplementary Figure S3B; Supplementary Sequence S6: BankIt accession number OR858756). The presence of linker L2 in Intron 3 of the mutant allele confirmed that editing had indeed occurred. The retention of the R106W mutation but insertion of linker L2 indicated that the editing vector recombined with the mutant allele and 5′ crossover event was located downstream of the mutation site but upstream of linker L2.

**Editing Outcome 3** accounted for 18% of recombination events and was characterized by the presence of genomic C SNP in Intron 2, linker L1, the wild type R106 codon, C SNP in Intron 3 and linker L2 (Figure 5B; Supplementary Figure S3B; Supplementary Sequence S7: BankIt accession number OR858757). This editing outcome resulted from a 5′ crossover event located upstream of L1 but downstream of the genomic C SNP in Intron 2. This HR event would result in either the correction of R106W mutation in the mutant allele or the retention of the wild type R106 in the wild type allele.

**Editing Outcome 4** also accounted for 18% of recombination events and was characterized by the presence of both the T SNP and linker L1 in Intron 2, the wild type R106 codon, the C SNP and linker L2 in Intron 3 (Figure 5B; Supplementary Figure S3B; Supplementary Sequence S8: BankIt accession number OR858758). Since the T SNP in Intron 2 is only present in the editing vector, we conclude that the 5′ crossover site was located upstream of the T SNP within the first 100 bp of the homology region. This outcome could have resulted from recombination of the editing vector with either the wild type or mutant allele.

Overall, 71% of the observed recombination events represented by Editing Outcomes 1, 3 and 4 contained wild type R106 codon (Figure 5; Supplementary Figure S3B). Similar to the editing outcomes observed in S134C cells, 29% of recombination events did not result in correction of R106W mutation due to the location of the 5′ crossover event downstream of mutation site. Moreover, we observed that there was a higher probability of correction when the variant nucleotides are located towards the center of the region of homology as opposed to the ends when the vector is homologous to the genomic sequence. These results are in agreement with a previously published study on the editing of the phenylalanine hydroxylase gene (Chen et al., 2020).

### Genome editing outcomes observed in heterozygous R282X female cells

**FIGURE 6:**
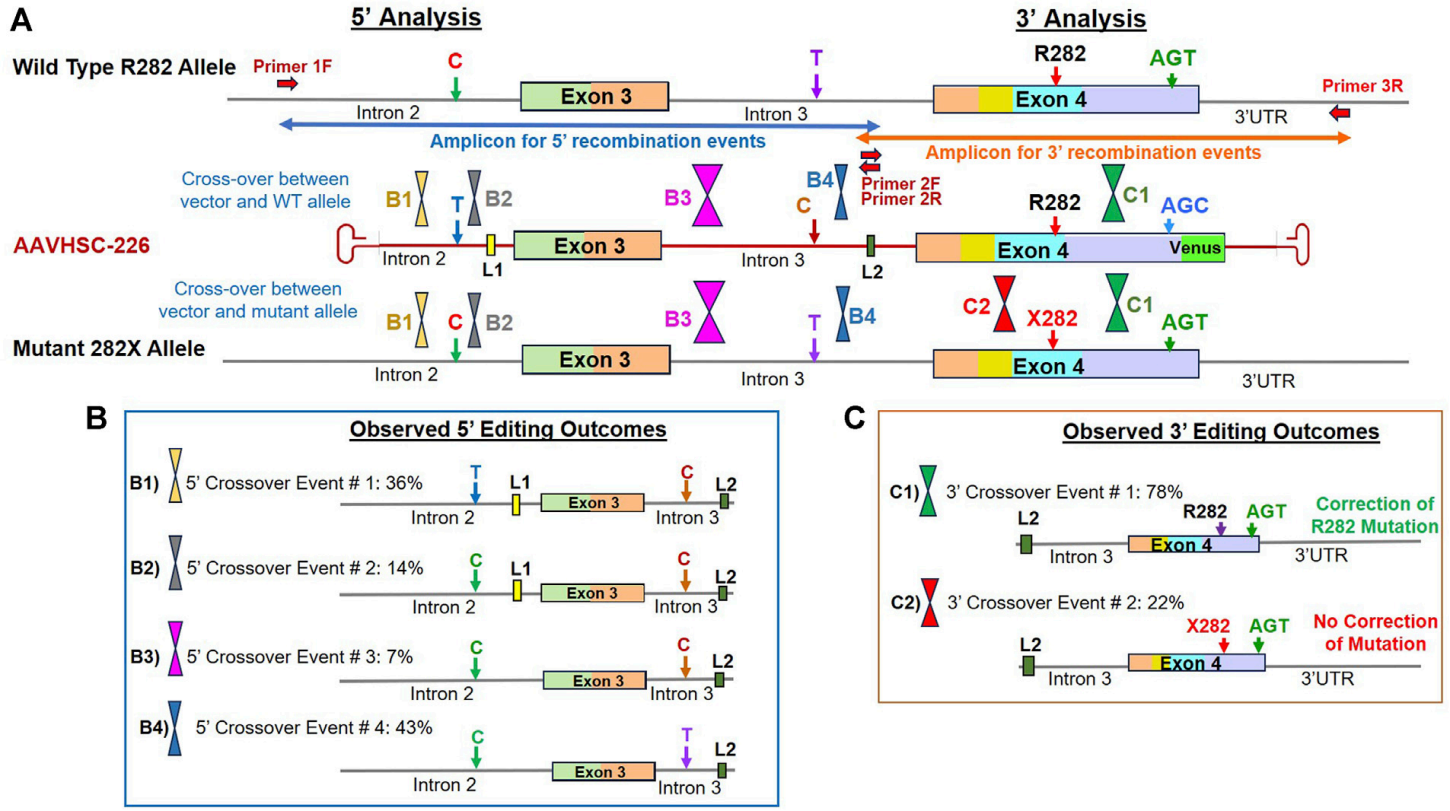
Sequence analysis of the edited MECP2 gene in heterozygous female R282X Rett syndrome cells. **(A)** Genome editing of the MECP2 gene in R282X cells. Shown are the WT R282 allele, AAVHSC-226 editing vector and the mutant 282X allele. Primers 1F and 3R are specific for chromosomal sequences external to the homology arms. Primers 2F and 2R are specific for linker L2. The crossover positions identified based on the presence or absence of markers in the edited genome and are depicted. **(B)** Observed 5′ editing outcomes. Four editing outcomes were identified at the 5′ end based on the presence or absence of specific SNPs and linkers as markers are shown. **(C)** Observed 3′ editing outcomes. Two editing outcomes identified at the 3′ end based on the SNPs and linker sequences as markers are shown.

Genome editing of R282X cells was analyzed following the evaluation of the 5′ and 3′ portions of the edited locus as described above, using primers specific for L2 in Intron 3 and chromosome-specific primers located outside the region of homology in either Intron 2 or the 3′ UTR. Genomic markers used to localize HR-related crossover events included: i) a C/T SNP in Intron 2, ii) the presence or absence of L1 in Intron 2, iii) a C/T SNP in Intron 3, iv) the presence or absence of L2 in Intron 3, v) the presence of either the WT R282 codon or the mutant R282X codon in Exon 4 and vi) a silent mutation for serine at codon 423 in Exon 4, where the vector encoded AGC while both the alleles of R282X genome encoded an AGT codon (Figure 6A). R282X genomic sequence contains 2 SNPs relative to the vector sequence. The genome contains a C SNP in Intron 2 and a T SNP in Intron 3 as opposed to the vector sequence, which contains T in Intron 2 and C in Intron 3 at the same position (Figure 6A).

#### 5′ editing outcomes observed in edited R282X fibroblasts

The 5′ editing outcomes of R282X cells were analyzed using the chromosome-specific forward primer, 1F which annealed to Intron 2 genomic sequence external to the region of homology and the L2 specific reverse primer (Supplementary Figure S4A). Evaluation of 5′ recombination outcomes in edited R282X cells revealed 4 distinct patterns of recombination (Figure 6B; Supplementary Figure S4B). In all cases, either the wild type or the mutant allele could have served as templates for HR.

**Editing Outcome B1** represented 36% of HR events which contained the T SNP in Intron 2, linker L1, C SNP and L2 in Intron 3 (Figure 6B; Supplementary Figure S4B; Supplementary Sequence S9: BankIt accession number OR858759). The insertion of these markers from the editing vector into the genome indicated successful editing and suggested that the 5′ crossover event occurred within the first 100 bp at the 5′ end of homology region.

**Editing Outcome B2** accounted for 14% of recombination events and contained the genomic C SNP and linker L1 in Intron 2, the C SNP and linker L2 in Intron 3, confirming successful editing (Figure 6B; Supplementary Figure S4B, Supplementary Sequence S10: BankIt accession number OR858760). The presence of the genomic C SNP and linker L1 in Intron 2 indicated that the 5′ crossover event occurred between these 2 markers.

**Editing Outcome B3** represented 7% of the observed recombination events. Here, the presence of the C SNP and linker L2 in Intron 3 confirmed successful editing as both the wild type and mutant alleles originally had the T SNP before editing (Figure 6B; Supplementary Figure S4B; Supplementary Sequence S11: BankIt accession number OR858761). The absence of linker L1 from the edited genome suggested that the 5′ crossover event occurred downstream of L1 but upstream of the C/T SNP in Intron 3.

**Editing Outcome B4** accounted for 43% of recombination events. Here, only linker L2 was found to be inserted into the genome following editing (Figure 6B; Supplementary Figure S4B; Supplementary Sequence S12: BankIt accession number OR858762). This suggested that the 5′ crossover occurred downstream of the C/T SNP in Intron 3 of genome but upstream of L2.

#### 3′ editing outcomes observed in edited R282X fibroblasts

The 3′ editing outcomes were analyzed by amplifying the 3′ portion of the edited genome using the forward primer 2F specific for linker L2 located within Intron 3 and the chromosome-specific reverse primer, 3R specific for 3′ UTR sequence downstream of the region of homology with the editing vector (Figure 6A; Supplementary Figure S5A). In this analysis, the T2A-Venus sequence containing TI amplicons were excluded as they were larger in size and did not amplify efficiently. The sequence analyses of the amplicon without T2A-Venus from 3′ portion of the edited region revealed 2 recombination patterns, C1 and C2 (Figure 6C; Supplementary Figure S5B). Both the recombination patterns observed retained the genomic AGT codon at the silent mutation S423 that is present in both the wild type and mutant alleles as compared with AGC in the editing vector. These results indicated that the 3′ crossover event occurred upstream of this codon.

**Editing Outcome C1** represented 78% of recombination events and contained both linker L2 and the wild type R282 codon (Figure 6C; Supplementary Figure S5B; Supplementary Sequence S13: BankIt accession number OR858763). The presence of linker L2 in the genome indicates that this allele was indeed edited as compared with the unedited wild type allele. However, it was not possible to determine whether the wild type or mutant allele had served as the template for HR.

**Edited outcome C2** accounted for 22% of recombination events and contained the mutant nucleotide encoding R282X in addition to linker L2 (Figure 6C; Supplementary Figure S5B; Supplementary Sequence S14: BankIt accession number OR858764). In this case, the mutant allele was indeed edited but mutation was not corrected. We reasoned that a 3′ crossover event located upstream of the R282X mutation could result in the absence of correction despite insertion of Linker L2, resulting in this recombination pattern.

In conclusion, sequence analyses of the editing outcomes observed in R282X cells following transduction with AAVHSC7 confirmed successful editing of the MECP2 gene. These results further indicated that due to the presence of significant homology between the editing vector and the target genome, crossover events associated with HR could be localized throughout the entire region of homology. We further concluded that correction of the MECP2 mutations was dependent upon the location of the crossover events. Similarly, insertion of the T2A-Venus ORF into the genome was also dependent upon the location of the crossover events with some corrective editing events resulting in the removal of the Venus cassette. Therefore, we conclude that the frequency of Venus expression observed by flow cytometry was an underrepresentation of the frequency of editing.

### HR-based genome editing was precise and seamless

On-target sequence analysis of genomic DNA from AAVHSC-transduced cells from 3 different Rett syndrome patients revealed that no indel mutations were identified in edited genomes (Supplementary Sequences S1–S14). Additionally, there was no evidence of insertion of AAV ITRs or other viral elements at the target site in the edited MECP2 gene. This is consistent with the HR-based DNA repair and previous reports of AAVHSC-based editing (Smith et al., 2018; Chen et al., 2020; Prout et al., 2023).

### Genome editing of a MECP2 deletion mutation restores expression

We next analyzed whether AAVHSC editing of the r.378_384del mutation in Exon 4 of MECP2 could rescue MECP2 expression in hemizygous male Rett cells (Coriell #GM21921). In GM21921 cells, a deletion at the Intron 3/Exon 4 splice acceptor site results in the creation of a new splice site. The resulting aberrantly spliced transcript has a deletion in Exon 4 which causes a frameshift mutation and introduces a premature termination codon resulting in a truncated MeCP2 protein that lacks the C-terminus amino acids (Schule et al., 2008). Transduction of GM21921 cells with the AAVHSC7-226 editing vector at MOI:150,000 resulted in Venus expression in 16% of cells based on flow cytometric analysis.

**FIGURE 7:**
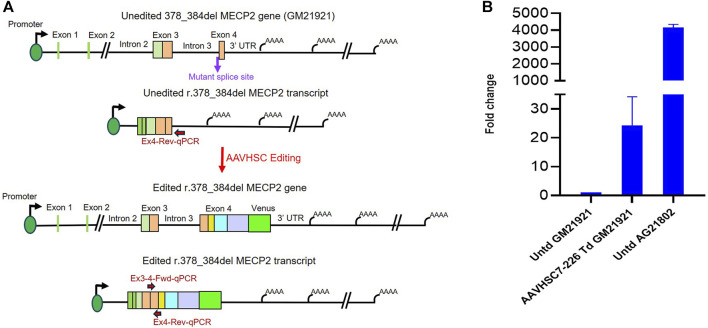
Rescue of MECP2 expression in edited hemizygous GM21921 fibroblasts. **(A)** Schematic showing the r.378_384del mutation in the genome and transcript of GM21921 cells before and after genome editing. The cells contain a deletion at the Intron 3/Exon 4 splice junction resulting in formation of a new splice site and a premature termination codon. AAVHSC7-226 editing restored the wild type splice junction and Exon 4 sequence in r.378_384del cells. The primers used for qRT-PCR are depicted. The forward primer is complementary to the Exon 3/Exon 4 splice junction in the wild-type spliced transcript, which is deleted in GM12921 cells. The reverse primer anneals to Exon 4 downstream of the mutant premature termination codon. **(B)** Quantitation of restoration of MECP2 expression after editing. Shown is the fold-change in MECP2 transcript levels in AAVHSC7-226 edited GM21921 cells (AAVHSC7-226 Td GM21921) and wild-type AG21802 cells (Untd AG21802) compared with unedited GM21921 cells (Untd GM21921). The fold change represents the average of 2 experiments, with 3 replicates each. Bars represent the standard deviation.

We assessed rescue of MECP2 expression by qRT-PCR after AAVHSC7-226 editing of GM21921 cells. MECP2 mRNA was analyzed using a forward primer complementary to the wild-type Exon 3/Exon 4 junction and a reverse primer complementary to a sequence in Exon 4 (Figure 7A). Results revealed that the correctly spliced mRNA was observed in edited but not in unedited cells. Figure 7 shows that a 24.18-fold increase in MECP2 transcript level was observed in edited GM21921 cells as compared to unedited controls (Figure 7B), indicating restoration of wild-type MECP2 transcription. MECP2 expression in the positive control wild-type AG21802 fibroblasts was ∼170-fold higher than in edited GM21921 cells, likely due to disparities in the genetic backgrounds of GM21921 and AG21802 cells. Since unedited cells do not contain the binding site for the forward primer due to the mutation, no amplification was observed. These results indicate that AAVHSC editing of GM21921 cells rescued MECP2 expression by restoring the deleted wild-type sequence.

#### Rescue of MeCP2 protein expression after AAVHSC editing

**FIGURE 8:**
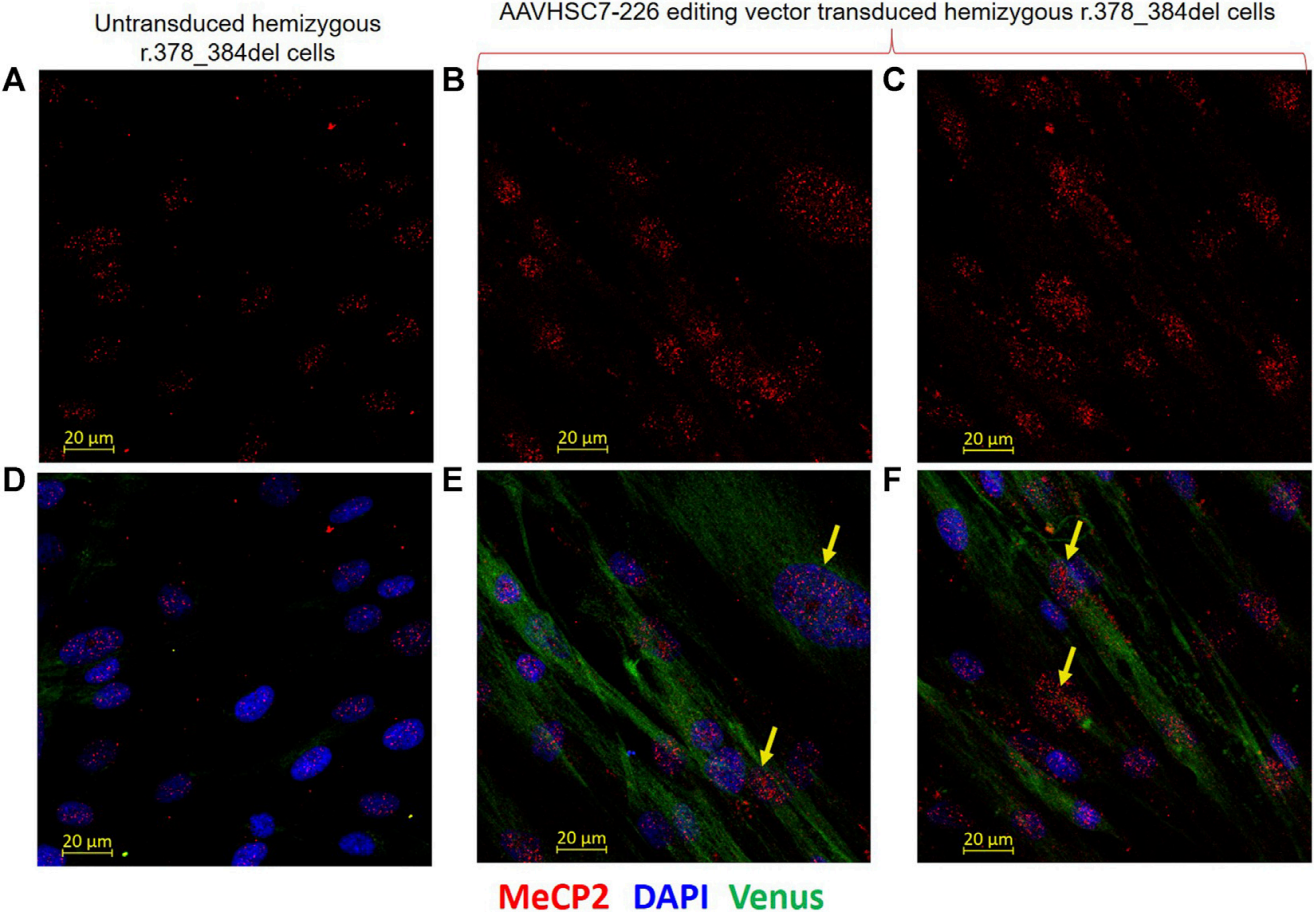
Restoration of MeCP2 expression in edited male hemizygous r.378_384del fibroblasts. Intranuclear expression of MeCP2 is shown in male hemizygous fibroblasts after transduction with the AAVHSC7-226 editing vector. **(A–C)** MECP2 staining in untransduced and transduced r.378_384del fibroblasts. **(A)** Unedited cells show no MeCP2 expression due to a deletion in C-Ter. **(B,C)** MeCP2 expression is observed in nuclei of r.378_384del fibroblasts 48 h after transduction with the AAVHSC7-226 editing vector. **(D)** No Venus expression is observed in untransduced cells. **(E, F)** show Venus expression in the cytoplasm after overlay of staining with an anti-GFP antibody. Also shown in **(D–F)** is the overlay with DAPI staining. **Yellow arrows**: examples of cells expressing both MeCP2 and Venus.

In order to evaluate restoration of MeCP2 protein expression, we performed immunofluorescence analysis of AAVHSC7 transduced GM21921 cells using a primary monoclonal antibody specific for the C-terminus of the MeCP2 protein and a secondary anti-rabbit Alexa Fluor-555 conjugated IgG (Figures 8A–C). Results demonstrated that AAVHSC7 editing of GM21921 cells restored specific MeCP2 expression in the nuclei of transduced cells (Figures 8B, C). Quantitative analysis of transduced GM21921 cells revealed that MECP2 expression was rescued in 29.2% cells after AAVHSC7 editing. The mean fluorescence intensity of MECP2 staining in edited cells was 1.9-fold higher than the background in unedited GM21921 cells (Supplementary Figure S6). In contrast, no MeCP2 expression was observed in untransduced control (Figure 8A), demonstrating that AAVHSC editing rescued MECP2 expression. Notably, MeCP2 expression in the nucleus appeared to be stippled, consistent with association with heterochromatin.

To further confirm whether restoration of MeCP2 expression coincided with Venus expression in edited cells, AAVHSC7 transduced GM21921 cells were stained *in situ* with both an anti-GFP antibody which cross-reacted with Venus and an anti-MECP2 (Figures 8D–F). Venus expression was clearly observed in the cytoplasm of transduced (Figures 8E,F), but not untransduced cells (Figure 8D). The coincident staining of Venus in the cytoplasm and MeCP2 in the nuclei of the same transduced cells clearly showed that AAVHSC7 edited cells specifically expressed both MeCP2 and Venus, confirming rescue and functional correction of the r.378_384del mutation. Thus, we conclude from these results that HR-based AAVHSC editing of the mutant MECP2 gene restores expression of MeCP2.

## Discussion

Here we showed for the first time that MECP2 mutations associated with Rett Syndrome can be corrected using the HR-based nuclease-free AAVHSC genome editing platform. Editing was demonstrated by Venus expression following targeted insertion of a promoterless cassette and by sequence analyses of edited genomes from 3 different Rett syndrome patient-derived cells. Importantly, MeCP2 expression was restored in edited cells, indicating that AAVHSC genome editing successfully rescues MECP2 mutations. The AAVHSC MECP2 editing vector used for these studies corrected mutations located in Exons 3 and 4 of the MECP2 gene, which harbor over 95% of pathogenic mutations associated with Rett syndrome.

Importantly, in our editing strategy, all native regulatory signals for physiologic expression of MECP2 are retained. These include the regulatory elements in the full-length promoter as well as in the 3′ UTR, including native miRNA binding sites. At 1 kb and 8.5 kb respectively, the entire promoter region and the 3′ UTR are too large to fit in AAV gene transfer vectors. Therefore, we reasoned that simply correcting mutations in the MECP2 gene would leave *in situ* all defined and yet undefined chromosomal regulatory elements, while reverting the mutant coding sequences back to wild type (Supplementary Sequence S3: BankIt accession number OR858753). This would maintain the stringent regulation required for physiologic expression of MeCP2. Notably, our approach is neither limited by the coding capacity of AAV vectors nor does it require prior detailed knowledge of the complete regulatory pathways for MeCP2. All chromosomal elements necessary for highly stringent regulation of MeCP2 are preserved resulting in physiologic expression of MeCP2, which is critical for global regulation of thousands of genes.

The MECP2 editing vector used in this study was fully homologous to the wild type sequence spanning from within MECP2 Intron 2 to part of the 3′ UTR. The only additional elements inserted into the genome consisted of a promoterless Venus ORF downstream of the MECP2 Exon 4 and 2 linkers in Introns 2 and 3. AAVHSC-mediated editing of primary fibroblasts and immortalized B-LCLs from Rett syndrome patients revealed Venus expression in edited cells following targeted insertion of the Venus ORF immediately downstream of Exon 4. Venus expression in edited cells was driven by the chromosomal MECP2 promoter and served as a surrogate marker for MECP2 expression. Venus expression was observed to be directly proportional to the MOI of the editing vector, confirming our previous observations at the AAVS1 locus (Smith et al., 2018). Comparison of different AAVHSC serotypes also confirmed our previous observations on the hierarchy of editing efficiencies between AAVHSC capsids (Smith et al., 2018). Successful editing was observed in all Rett syndrome cells tested, including those with mutations in the MBD and TRD domains.

We have previously shown that AAVHSC editing utilizes a BRCA2-dependent HR mechanism (Smith et al., 2018; Bijlani et al., 2021). Here, replacement of target genomic sequences with a vector-encoded sequence requires a 5′ and a 3′ crossover event at the beginning and the end of the target/insert sequence. The insertion of SNPs and linkers allowed localization of crossover events that resulted in specific sequence outcomes. Analysis of the edited MECP2 gene in Rett syndrome cells indicated that due to the extensive (3.5 kb) homology between the editing moiety in the vector and the target region, crossover events for HR occurred throughout the region of homology. Hemizygous male cells contain a single copy of the X-linked MECP2 gene which served as the template for HR. Nearly half of recombination events were found to result in correction of the S134C mutation to encode serine, while the remaining edited genomes retained the mutant cysteine at residue 134. In the latter instances, evidence of editing was obtained from the insertion of the linker into the intron. Our results indicated that the S134C mutation site was excluded in 56% of recombination events.

In contrast, heterozygous female cells have 2 alleles of the MECP2 gene. Here either the mutant or the wild type allele or both could serve as templates for HR and editing could be either monoallelic or biallelic. In R106W cells, where the pathogenic mutation was located in MBD in Exon 3, 71% of all edited genomes analyzed had the wild type arginine at residue 106, while 29% retained the mutant tryptophan. Sequence analysis of edited heterozygous R282X cells where the mutation is located in TRD in Exon 4, revealed that 78% of edited genomes had the wild type arginine at amino acid 282, while 22% had the premature terminator. For the heterozygous female Rett syndrome cells analyzed, it was not possible to determine whether the mutant or the wild type allele served as the template for HR since the sequence outcome after editing was identical for both alleles. Long range sequence identification of allele-specific SNPs located outside the target region may allow identification of whether the mutant or the wild type allele served as the template for editing.

We conclude that when fully homologous large HR donor elements/editing moieties were used, crossover events were located throughout the region of homology. Each homologous recombination event is mediated by a 5′ and a 3′ crossover. When these crossover events flank the MECP2 mutation site, the mutation was found to be corrected. On the other hand, when both crossover events occurred either upstream or downstream of the mutation, the mutation was not corrected. This suggests that editing efficiencies may be increased by altering the design of editing elements to ensure that every recombination event results in the correction of mutations.

The editing outcomes as determined by sequence analyses also suggested that editing efficiencies estimated from Venus expression were likely an under-representation. In instances where the 3′ crossover events occurred upstream of the Venus ORF, edited genomes did not contain the Venus ORF, despite successful editing. This would be similar to situations where the mutation was retained despite successful editing. Nonetheless, substantial levels of mutation correction were observed at the sequence level. Whether this is sufficient for *in vivo* therapeutic efficacy awaits testing in disease models.

Sequence analyses of the edited Rett syndrome cells revealed that the AAVHSC editing of the MECP2 locus was precise with no evidence of indel mutations or insertion of AAV ITRs. This confirms previous reports on the on-target accuracy of AAVHSC editing where no on-target indel mutations or AAV ITR insertions were detected at either the human AAVS1, the murine Rosa26 loci (Smith et al., 2018), the human and murine phenylalanine hydroxylase loci (Chen et al., 2020; Prout et al., 2023). This is in contrast to the higher frequency of indel mutations noted following CRISPR-Cas9 mediated correction of the T158M MECP2 mutations in patient-derived cells (Croci et al., 2020).

Off-target genome-wide mutagenesis is also a potential concern with nuclease-based editing platforms. The use of CRISPR-Cas9 has been shown to result in large structural changes including chromosome loss, translocations and deletions due to promiscuous cleavage at off-target sites (Fu et al., 2013; Pattanayak et al., 2013; Kuscu et al., 2014; Adikusuma et al., 2018; Kosicki et al., 2018; Tsuchida et al., 2023). Although at a lower frequency than Cas9, base and prime editors also resulted in large deletions and translocations (Fiumara et al., 2023). However, as opposed to nuclease-based editing platforms, AAVHSC editing did not result in any off-target effects *in vivo* (Prout et al., 2023), likely due to the absence of nucleases that inevitably result in off-target cleavage.

Notably, we demonstrated that AAVHSC editing rescued MECP2 mutations and restored MECP2 transcript and protein expression in hemizygous male cells bearing an Intron3/Exon 4 deletion, indicating the potential for restoration of physiologic MeCP2 expression after AAVHSC editing. Coexpression of MeCP2 in the nuclei and Venus in the cytoplasm of the same cells confirmed that AAVHSC editing resulted in correction at the protein level. The detection of Venus expression further confirmed correct in-frame targeted insertion of Venus ORF downstream of Exon 4.

Thus, AAVHSC HR-based genome editing platform has tremendous potential for the precise and accurate correction of pathogenic MECP2 mutations associated with Rett syndrome in patient-derived cells with no evidence of on-target mutations or ITR insertions. AAVHSC editing resulted in the successful rescue of MECP2 mutations and faithful restoration of the wild type genomic sequence and MeCP2 expression, suggesting *in vivo* therapeutic utility. Notably, the use of a fully homologous editing moiety precluded specification of precise locations for HR crossover events and resulted in a reduced frequency of mutation correction relative to total editing efficiencies. Novel modifications of the editing moiety and HR strategies will be required to overcome these differences in the editing versus correction efficiencies.

## Data Availability

The data presented in the study are deposited in NCBI BankIt repository, accession numbers OR858751, OR858752, OR858753, OR858754, OR858755, OR858756, OR858757, OR858758, OR858759, OR858760, OR858761, OR858762, OR858763, OR858764.
