# Supplementary figures and images for: Nuclease-free precise genome editing corrects MECP2 mutations associated with Rett syndrome
Source: Front Genome Ed. 2024 Mar 1;6:1346781. doi: 10.3389/fgeed.2024.1346781 (PMC10940404; doi:10.3389/fgeed.2024.1346781)

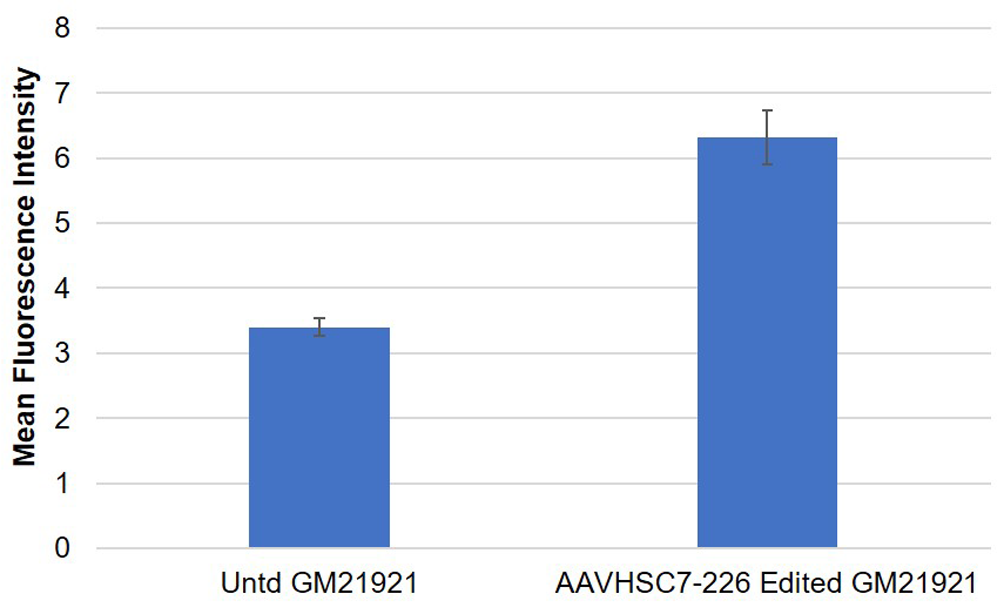

Supplement: Supplementary file 1 [file Image6.TIF]

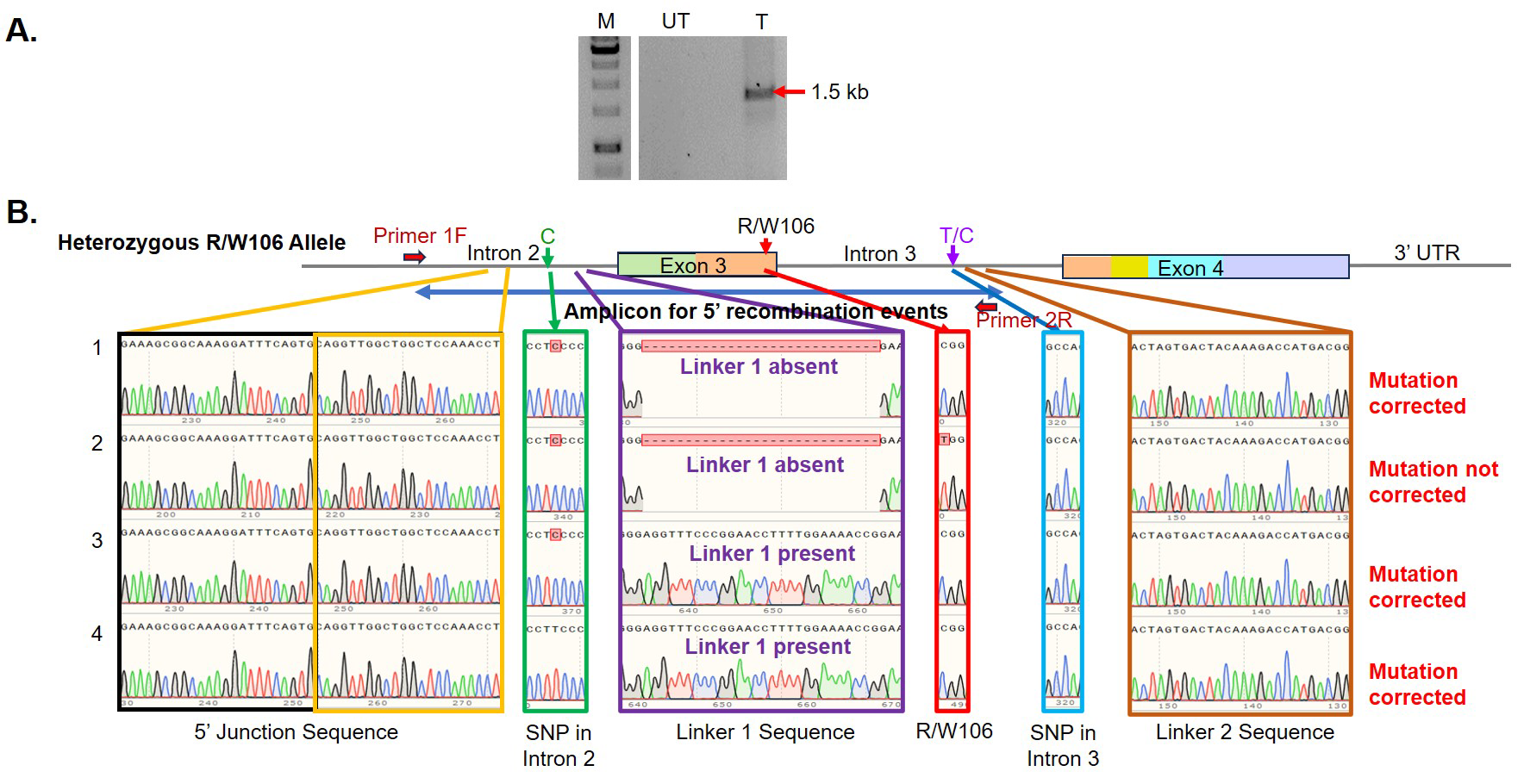

Supplement: Supplementary file 2 [file Image3.TIF]

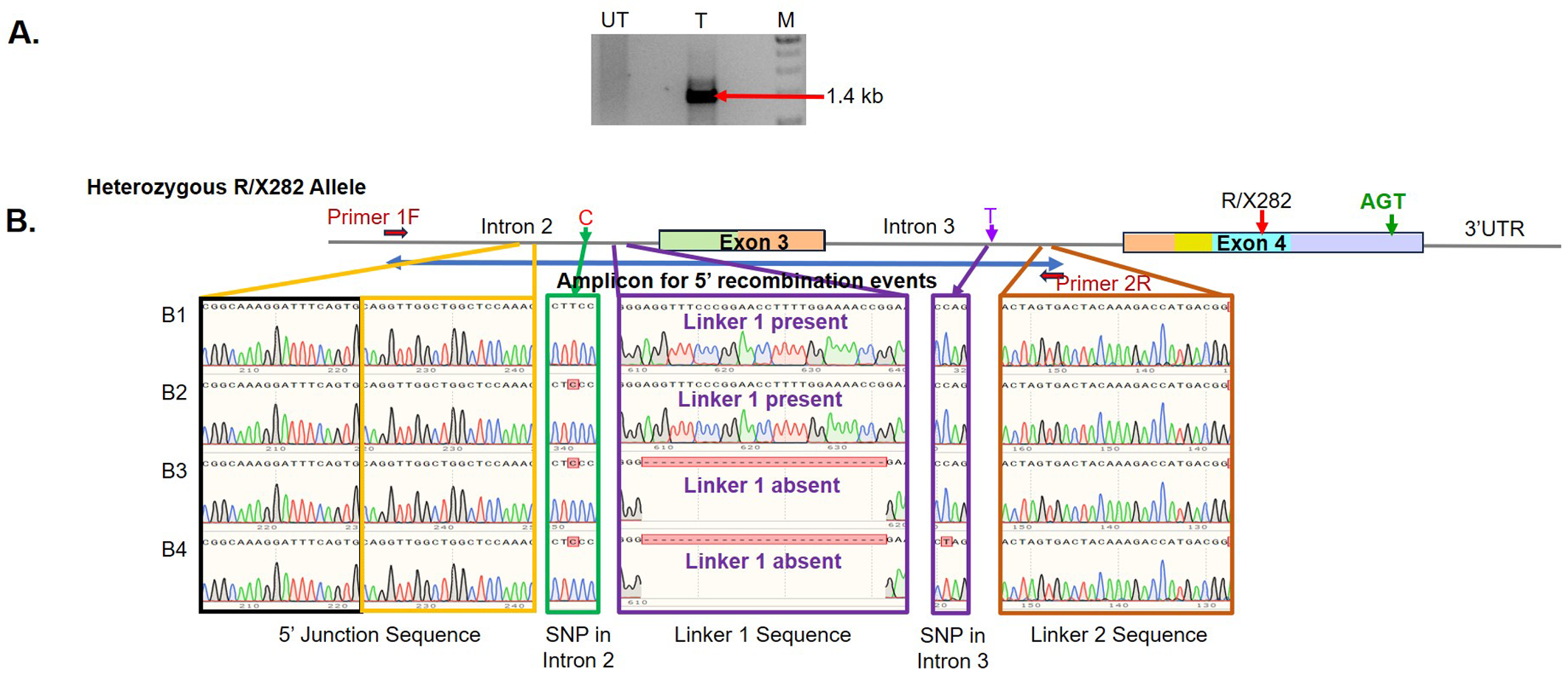

Supplement: Supplementary file 3 [file Image4.TIF]

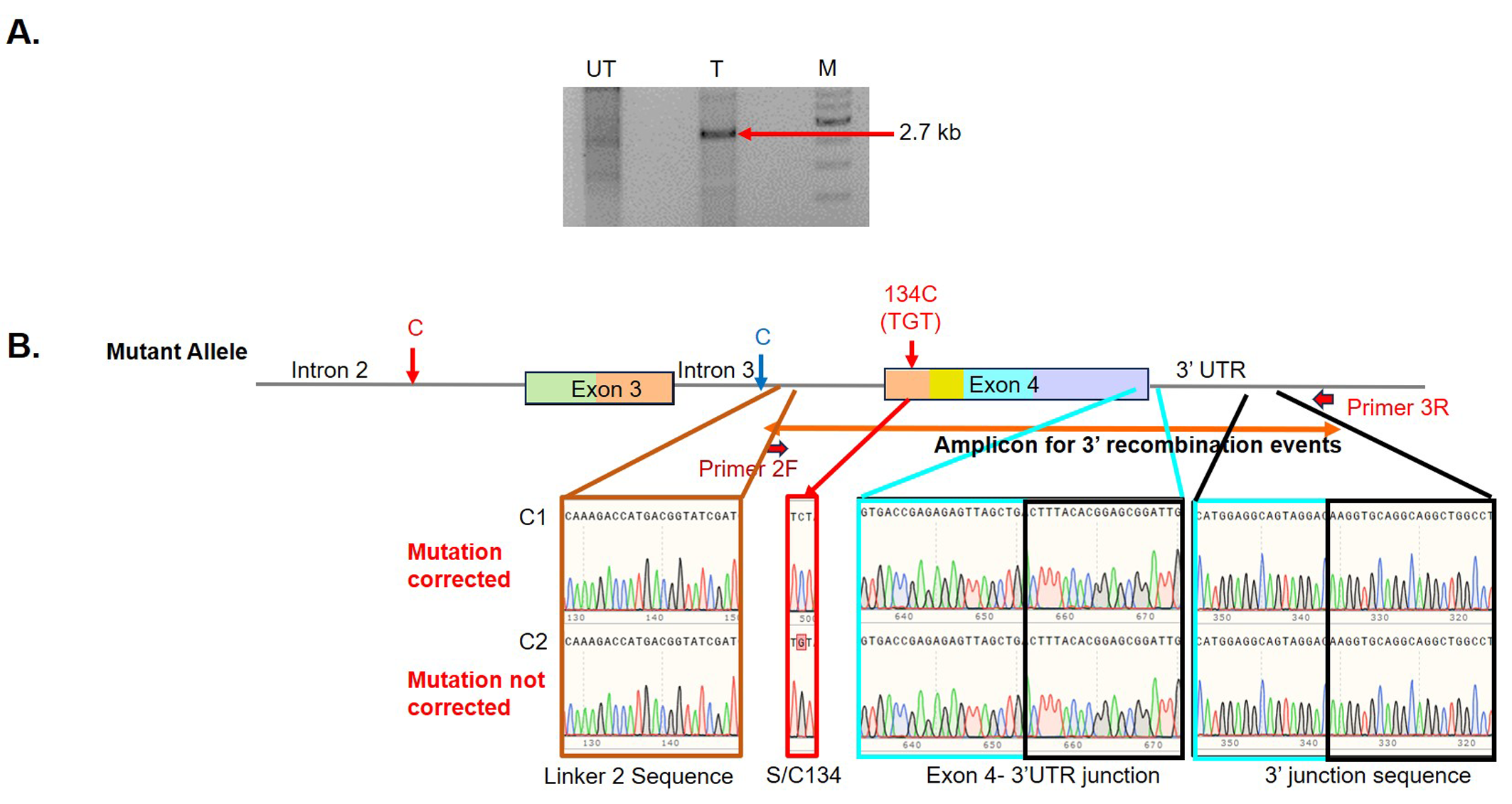

Supplement: Supplementary file 4 [file Image2.TIF]

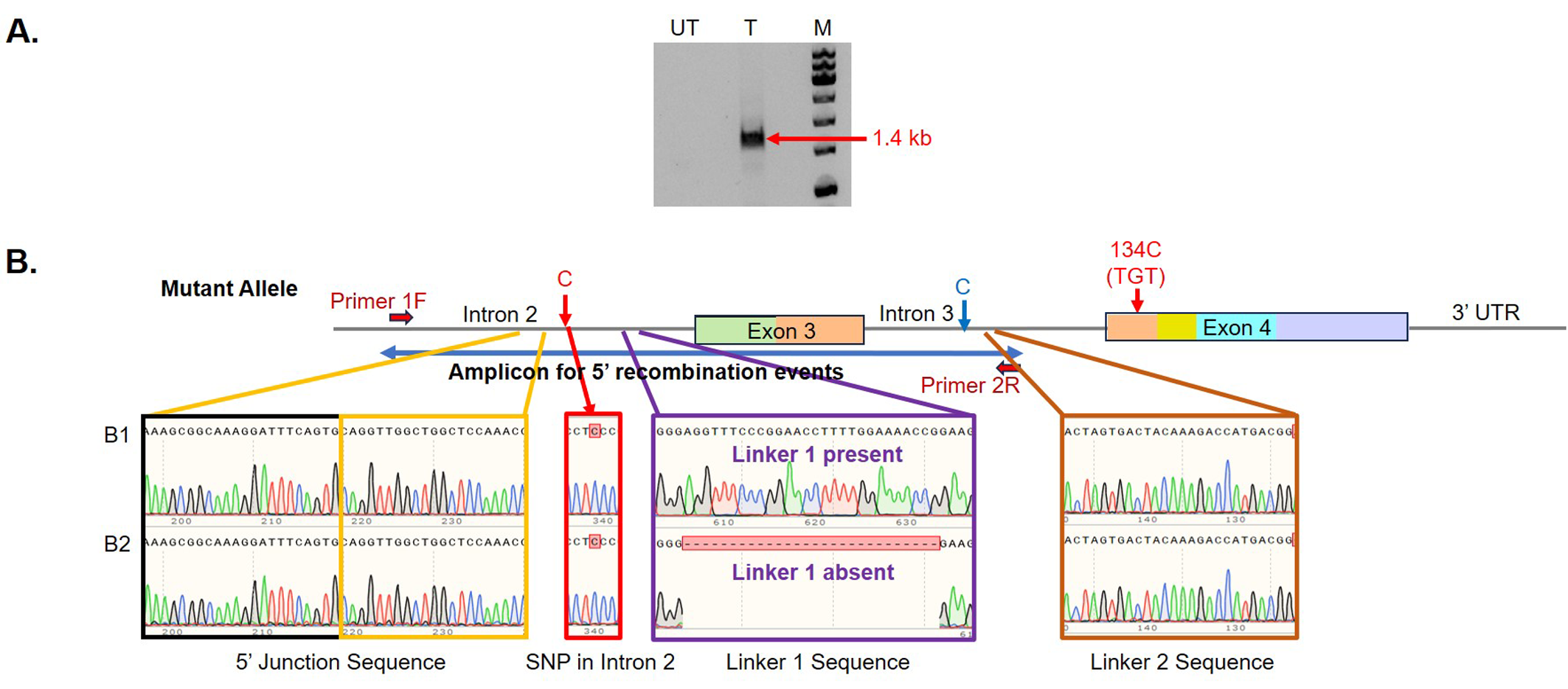

Supplement: Supplementary file 5 [file Image1.TIF]

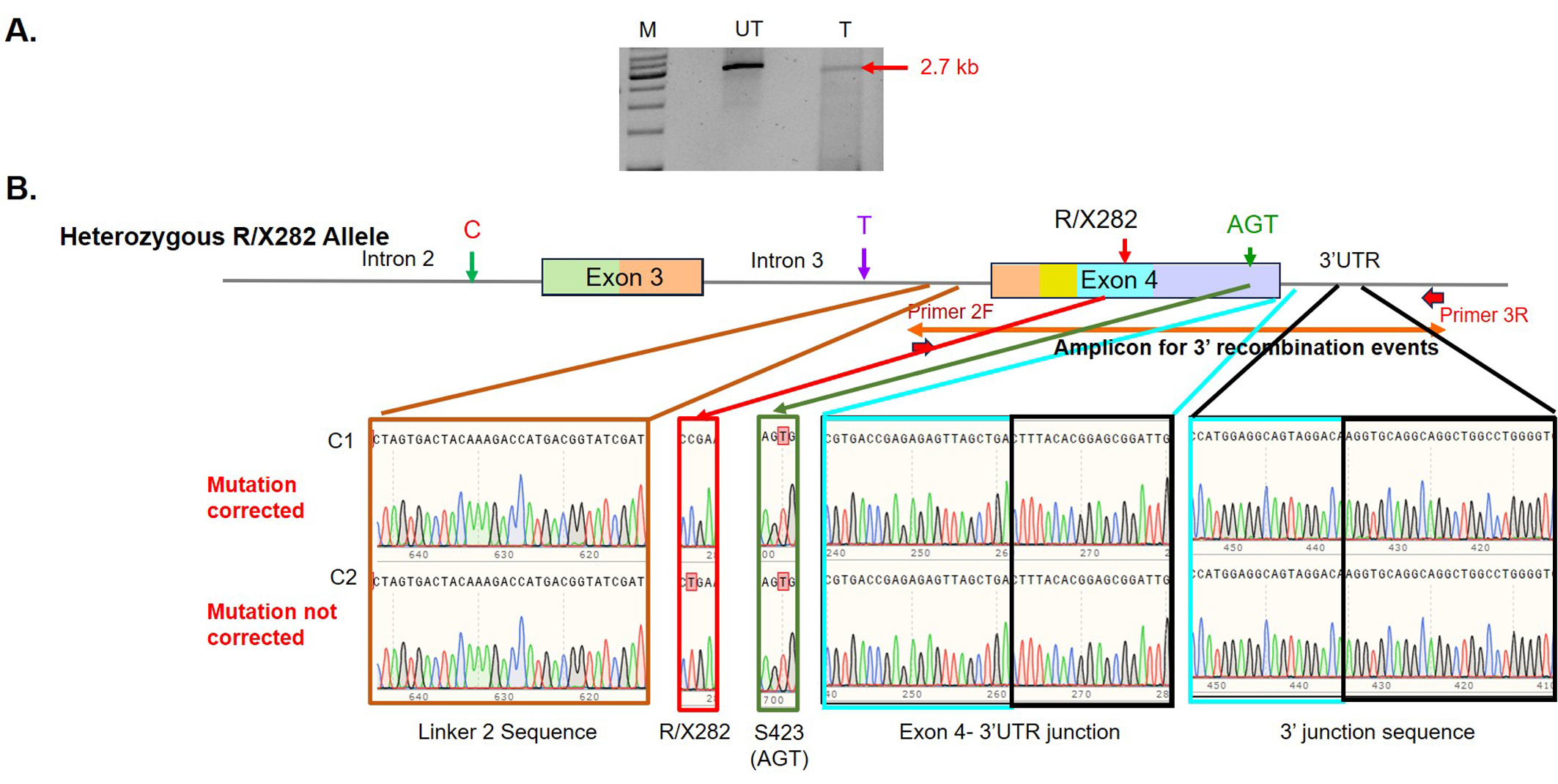

Supplement: Supplementary file 7 [file Image5.TIF]
