# Supplementary material for: Nuclease-free precise genome editing corrects MECP2 mutations associated with Rett syndrome
Source: Front Genome Ed. 2024 Mar 1;6:1346781. doi: 10.3389/fgeed.2024.1346781 (PMC10940404; doi:10.3389/fgeed.2024.1346781)
Supplement: Supplementary file 6 [file DataSheet1.pdf]

Supplementary material

Table S1. List of primers used in the study.

Primer	Sequence (5'→3')	Description
SCO231	atgcatGTCCTACTGCCTCCATGGCC	Primers used for cloning of AAVHSC-226 editing vector
SCO232	gcggccgcatatgCAGGTTGGCTGGCTCCAAAC	
SCO221	GAGGTTTCCCGGAACCTTTTGGAAAACC	
SCO222	CGGTTTTCCAAAAGGTTCCGGGAAACCT	
AP1 MecP2	ACTAGTGACTACAAAGACCATGACGGTATCGATCGTACCTGAGTTCAAACTTG	
AP2 MecP2	ATCGATACCGTCATGGTCTTTGTAGTCACTAGTGTGCTCAGTCTCTCCAGG	
qVenus-Fwd	ctgctgcccgacaacca	Primers used for qPCR to titer vector
qVenus-Rev	tgtgatcgcgcttctcgtt	
qVenus-probe	56-FAM/AGCTACCAGTCCGCCCTGAGCAAAGA/3IABkFQ	
Primer 1F	GCTCACAGTAAGGATGCCTAGATG/ GGGTTACCTGTTGTCAAGATAAGGAAAC	Primers used for TI assay
Primer 2R	CCGTCATGGTCTTTGTAGTCACTAG	
Primer 2F	CTAGTGACTACAAAGACCATGACGG	
Primer 3R	AGAAGTGAAAGGATGAAATGAACAAAAG	
Primer 4F	CAAAGACCATGACGGTATCGATC	
Primer 4R	GGGAACATGAAGACTCAATAGTGC	
Ex3-4-Fwd-qPCR	GTGTATTTGATCAATCCCCAGGG	Primers used for MECP2 qRT-PCR
Ex4-Rev-qPCR	GGCTTCTTAGGTGGTTTCTGCTC	
Ex4-probe-qPCR	56-FAM/CTCCCTCTCCCAGTTACCGTGAAGTCAA/3IABkFQ	

Sequences S1-S14 listed below have the following features highlighted:

Gray highlighted text: Forward primer (Primer 1F) upstream of 5' homology arm

Yellow highlighted text: Reverse primer (Primer 2F/Primer 2R) corresponding to L2

Blue highlighted text: Reverse primer (Primer 3R) downstream of 3' homology arm

Underlined seq: Marks the junction between the genomic DNA and vector homology arms

Pink bold font: L1 sequence inserted in MECP2 Intron 2

Purple bold font: L2 sequence inserted in MECP2 Intron 3

Red bold text: SNPs between the MECP2 sequence on vector and genome in Intron 2 and Intron 3

Green bold font: Codon corresponding to wild type or mutant amino acid

Sequence S1. Consensus sequence of Edited Outcome B1 in S134C cells represented in Figure 4B.

GGGTTACCTGTTGTCAAGATAAGGAAACTGAAGCACAGAATGCTGAGGTCATTTGCT
GGGTTCATGTTTGGAAAGCGGCAAAGG<u>ATTTCAGTGCAGGTTGGCT</u>GGCTCCAAACC
TGTGTGTGCTTTCCATGACACTGTACTGTGTGCCTCATTGAGCCTCATTCTAGAAAAC
CAAAAACACACCCAAGGCCCGGCCTTCACAAAGGAGACCCCT**C**CCCCATTTGGCTC
CCTTTCCAGCAGTCGACGGCCTCTTGTCAGCCATCGAGCCCAGAGTCCCTTGAAGTG
CGACTCATGCTGGGGTGGTATGCTCAGGAGCCGCAGTGTTTCCGCTCAGAGGAAAG
GGCTCTGATTCTCCTGCAGTGCTAGGAGACTTGTGGGTGGCCACAGTGCAGGTCAGG
CACACCGGCCAGCACCACCCACAGCCCAAATTCCTAAAGAAATATTTGGGTCCCAG
CTTGGCCCGAGTCTCTGTTGTCCTGGGGAAGGACATCAAGATCTGAGTGTATGATGG
CCTGGGGCCTTGCATGTGGTGGGGGTCCAAGCCTGCCTCTGCTCACTTGTTCTGCAG
ACTGGCATGTTCTCTGTGATACTTACATACTTGTTTAACACTTCAGGGAAGAAAAGT
CAGAAGACCAGGACCTCCAGGGCCTCAAGGACAAACCCCTCAAGTTTAAAAAGGTG
AAGAAAGATAAGAAAGAAGAGAAAGAGGGCAAGCATGAGCCCGTGCAGCCATCAG
CCCACCACTCTGCTGAGCCCGCAGAGGCAGGCAAAGCAGAGACATCAGAAGGGTCA
GGCTCCGCCCCGGCTGTGCCGGAAGCTTCTGCCTCCCCCAAACAGCGGCGCTCCATC
ATCCGTGACCGGGGACCCATGTATGATGACCCCACCCTGCCTGAAGGCTGGACACG
GAAGCTTAAGCAAAGGAAATCTGGCCGCTCTGCTGGGAAGTATGATGTGTATTTGAT
CAAGTAAGTAAGAGCAACTCCTATCTCTACAGGGCAGGGAGGGCAGGGACAAGGAT
CCCTCATGGAGCAGGAAAATGTATGTGCCCAGGGTGGGGTCGGGGGGAACATAAAC
AATGAACACTGAGACCAGGTGTGCTTGAAATGACCGTGTACAGAGGTCGCTGCCCT
GAGTGGGAAGTTCTCAAGGTAGCAGGCCCTCTATCCTCTCCACACCTCAAGTCTTTA
TCTGGGGATGGAATAGCTGCGGAAGCAGAGGAACTTGCAGAGC**C**AGGGGTTCAGAG
GGGTGAAGAAGCATGTTTCAGTTCTGCCTTTTAAATGATCCCAAAAAGGTTAGCAGT
TTTCAAATGACATTTGCAGACAGCCTCATTTAATTCCATGAGAAGGGTGAGCAAAGG
ATTATCTTGTTGAAACTGATTCCTGGAGAGACTGAGCACA**ACTAGTGACTACAAAGA
CCATGACGG**

Sequence S2. Consensus sequence of Edited Outcome B2 in S134C cells represented in Figure 4B.

GGGTTACCTGTTGTCAAGATAAGGAAACTGAAGCACAGAATGCTGAGGTCATTTGCT
GGGTTCATGTTTGGAAAGCGGCAAAGGATTTCAGTGCAGGTTGGCTGGCTCCAAACC
TGTGTGTGCTTTCCATGACACTGTACTGTGTGCCTCATTGAGCCTCATTCTAGAAAAC
CAAAAACACACCCAAGGCCCGGCCTTCACAAAGGAGACCCCTCCCCCATTTGGCTC
CCTTTCCAGCAGTCGACGGCCTCTTGTCAGCCATCGAGCCCAGAGTCCCTTGAAGTG
CGACTCATGCTGGGGTGGTATGCTCAGGAGCCGCAGTGTTTCCGCTCAGAGGAAAG
GGCTCTGATTCTCCTGCAGTGCTAGGAGACTTGTGGGTGGCCACAGTGCAGGTCAGG
CACACCGGCCAGCACCACCCACAGCCCAAATTCCTAAAGAAATATTTGGGTCCCAG
CTTGGCCCGAGTCTCTGTTGTCCTGGGAGGTTTCCCGGAACCTTTTGGAAAACCG
GAAGGACATCAAGATCTGAGTGTATGATGGCCTGGGGCCTTGCATGTGGTGGGGGT
CCAAGCCTGCCTCTGCTCACTTGTTCTGCAGACTGGCATGTTCTCTGTGATACTTACA
TACTTGTTTAACACTTCAGGGAAGAAAAGTCAGAAGACCAGGACCTCCAGGGCCTC
AAGGACAAACCCCTCAAGTTTAAAAAGGTGAAGAAAGATAAGAAAGAAGAGAAAG
AGGGCAAGCATGAGCCCGTGCAGCCATCAGCCCACCACTCTGCTGAGCCCGCAGAG
GCAGGCAAAGCAGAGACATCAGAAGGGTCAGGCTCCGCCCCGGCTGTGCCGGAAGC
TTCTGCCTCCCCCAAACAGCGGCGCTCCATCATCCGTGACCGGGGACCCATGTATGA
TGACCCCACCCTGCCTGAAGGCTGGACACGGAAGCTTAAGCAAAGGAAATCTGGCC
GCTCTGCTGGGAAGTATGATGTGTATTTGATCAAGTAAGTAAGAGCAACTCCTATCT
CTACAGGGCAGGGAGGGCAGGGACAAGGATCCCTCATGGAGCAGGAAAATGTATGT
GCCCAGGGTGGGGTCGGGGGGAACATAAACAATGAACACTGAGACCAGGTGTGCTT
GAAATGACCGTGTACAGAGGTCGCTGCCCTGAGTGGGAAGTTCTCAAGGTAGCAGG
CCCTCTATCCTCTCCACACCTCAAGTCTTTATCTGGGGATGGAATAGCTGCGGAAGC
AGAGGAACTTGCAGAGCCAGGGGTTCAGAGGGGTGAAGAAGCATGTTTCAGTTCTG
CCTTTTAAATGATCCCAAAAAGGTTAGCAGTTTTCAAATGACATTTGCAGACAGCCT
CATTTAATTCCATGAGAAGGGTGAGCAAAGGATTATCTTGTTGAAACTGATTCCTGG
AGAGACTGAGCACA**CTAGTGACTACAAAGACCATGACGG**

Sequence S3. Consensus sequence of Edited Outcome C1 in S134C cells represented in Figure 4C.

CAAAGACCATGACGGTATCGATCGTACCTGAGTTCAAACTTGGGAATGTTCTAGAT
GGTGACTCAGGCCCAGGCACCAACCAGCAGAATGGGCCTCAGCCTGACAACCCTTC
TGTACCAGGCCTGACTCTTTGGTTGCTGAACTTTGGAGAGGCCTGGGGGGGTCAGCG
GCAGGCAGACGAGTGAGTGGCTTTGGTGACAGGTCCTCAGGGGCAGCCAGGCAGTG
TGACTCTCGTTCAATAGTAACGTTTGTCAGAGCGTTGTCACCACCATCCGCTCTGCCC
TATCTCTGACATTGCTATGGAGAGCCTCTAATTGTTCCTTGTGTCTTTCTGTTTGTCCC
CACAGTCCCCAGGGAAAAGCCTTTCGC**TCT**AAAGTGGAGTTGATTGCGTACTTCGAA
AAGGTAGGCGACACATCCCTGGACCCTAATGATTTTGACTTCACGGTAACTGGGAGA
GGGAGCCCCTCCCGGCGAGAGCAGAAACCACCTAAGAAGCCCAAATCTCCCAAAGC
TCCAGGAACTGGCAGAGGCCGGGGACGCCCCAAAGGGAGCGGCACCACGAGACCC
AAGGCGGCCACGTCAGAGGGTGTGCAGGTGAAAAGGGTCCTGGAGAAAAGTCCTGG

GAAGCTCCTTGTCAAGATGCCTTTTCAAACTTCGCCAGGGGGCAAGGCTGAGGGGG
GTGGGGCCACCACATCCACCCAGGTCATGGTGATCAAACGCCCCGGCAGGAAGCGA
AAAGCTGAGGCCGACCCTCAGGCCATTCCCAAGAAACGGGGCCGAAAGCCGGGGA
GTGTGGTGGCAGCCGCTGCCGCCGAGGCCAAAAAGAAAGCCGTGAAGGAGTCTTCT
ATCCGATCTGTGCAGGAGACCGTACTCCCCATCAAGAAGCGCAAGACCCGGGAGAC
GGTCAGCATCGAGGTCAAGGAAGTGGTGAAGCCCCTGCTGGTGTCCACCCTCGGTG
AGAAGAGCGGGAAAGGACTGAAGACCTGTAAGAGCCCTGGGCGGAAAAGCAAGGA
GAGCAGCCCCAAGGGGCGCAGCAGCAGCGCCTCCTCACCCCCCAAGAAGGAGCACC
ACCACCATCACCACCACTCAGAGTCCCCAAAGGCCCCCGTGCCACTGCTCCCACCCC
TGCCCCCACCTCCACCTGAGCCCGAGAGCTCCGAGGACCCCACCAGCCCCCCTGAGC
CCCAGGACTTGAGCAGCAGCGTCTGCAAAGAGGAGAAGATGCCCAGAGGAGGCTC
ACTGGAGAGCGACGGCTGCCCCAAGGAGCCAGCTAAGACTCAGCCCGCGGTTGCCA
CCGCCGCCACGGCCGCAGAAAAGTACAAACACCGAGGGGAGGGAGAGCGCAAAGA
CATTGTTTCATCCTCCATGCCAAGGCCAAACAGAGAGGAGCCTGTGGACAGCCGGA
CGCCCGTGACCGAGAGAGTTAGCTGACTTTACACGGAGCGGATTGCAAAGCAAACC
AACAAGAATAAAGGCAGCTGTTGTCTCTTCTCCTTATGGGTAGGGCTCTGACAAAGC
TTCCCGATTAACTGAAATAAAAAATATTTTTTTTCTTTCAGTAAACTTAGAGTTTCG
TGGCTTCAGGGTGGGAGTAGTTGGAGCATTGGGGATGTTTTCTTACCGACAAGCAC
AGTCAGGTTGAAGACCTAACCAGGGCCAGAAGTAGCTTTGCACTTTTCTAAACTAGG
CTCCTTCAACAAGGCTTGCTGCAGATACTACTGACCAGACAAGCTGTTGACCAGGCA
CCTCCCCTCCCGCCCAAACCTTTCCCCCATGTGGTCGTTAGAGACAGAGCGACAGAG
CAGTTGAGAGGACACTCCCGTTTTCGGTGCCATCAGTGCCCCGTCTACAGCTCCCCC
AGCTCCCCCCACCTCCCCCACTCCCAACCACGTTGGGACAGGGAGGTGTGAGGCAG
GAGAGACAGTTGGATTCTTTAGAGAAGATGGATATGACCAGTGGCTATGGCCTGTG
CGATCCCACCCGTGGTGGCTCAAGTCTGGCCCCACACCAGCCCCAATCCAAAACTGG
CAAGGACGCTTCACAGGACAGGAAAGTGGCACCTGTCTGCTCCAGCTCTGGCATGG
CTAGGAGGGGGGAGTCCCTTGAACTACTGGGTGTAGACTGGCCTGAACCACAGGAG
AGGATGGCCCAGGGTGAGGTGGCATGGTCCATTCTCAAGGGACGTCCTCCAACGGG
TGGCGCTAGAGGCCATGGAGGCAGTAGGACAAGGTGCAGGCAGGCTGGCCTGGGGT
CAGGCCGGGCAGAGCACAGCGGGGTGAGAGGGATTCCTAATCACTCAGAGCAGTCT
GTGACTTAGTGGACAGGGGAGGGGGCAAAGGGGGAGGAGAAGAAAATGTTCTTCC
AGTTACTTTCCAATTCTCCTTTAGGGACAGCTTAGAATTATTTGCACTATTGAGTCTT
CATGTTCCC

Sequence S4. Consensus sequence of Edited Outcome C2 in S134C cells represented in Figure 4C.

CAAAGACCATGACGGTATCGATCGTACCTGAGTTCAAACTTGGGAATGTTCTAGAT
GGTGACTCAGGCCCAGGCACCAACCAGCAGAATGGGCCTCAGCCTGACAACCCTTC
TGTACCAGGCCTGACTCTTTGGTTGCTGAACTTTGGAGAGGCCTGGGGGGGTCAGCG
GCAGGCAGACGAGTGAGTGGCTTTGGTGACAGGTCCTCAGGGGCAGCCAGGCAGTG
TGACTCTCGTTCAATAGTAACGTTTGTCAGAGCGTTGTCACCACCATCCGCTCTGCCC
TATCTCTGACATTGCTATGGAGAGCCTCTAATTGTTCCTTGTGTCTTTCTGTTTGTCCC
CACAGTCCCCAGGGAAAAGCCTTTCGC**TGT**AAAGTGGAGTTGATTGCGTACTTCGA

AAAGGTAGGCGACACATCCCTGGACCCTAATGATTTTGACTTCACGGTAACTGGGAG
AGGGAGCCCCTCCCGGCGAGAGCAGAAACCACCTAAGAAGCCCAAATCTCCCAAAG
CTCCAGGAACTGGCAGAGGCCGGGGACGCCCCAAAGGGAGCGGCACCACGAGACC
CAAGGCGGCCACGTCAGAGGGTGTGCAGGTGAAAAGGGTCCTGGAGAAAAGTCCTG
GGAAGCTCCTTGTCAAGATGCCTTTTCAAACTTCGCCAGGGGGCAAGGCTGAGGGG
GGTGGGGCCACCACATCCACCCAGGTCATGGTGATCAAACGCCCCGGCAGGAAGCG
AAAAGCTGAGGCCGACCCTCAGGCCATTCCCAAGAAACGGGGCCGAAAGCCGGGG
AGTGTGGTGGCAGCCGCTGCCGCCGAGGCCAAAAAGAAAGCCGTGAAGGAGTCTTC
TATCCGATCTGTGCAGGAGACCGTACTCCCCATCAAGAAGCGCAAGACCCGGGAGA
CGGTCAGCATCGAGGTCAAGGAAGTGGTGAAGCCCCTGCTGGTGTCCACCCTCGGT
GAGAAGAGCGGGAAAGGACTGAAGACCTGTAAGAGCCCTGGGCGGAAAAGCAAGG
AGAGCAGCCCCAAGGGGCGCAGCAGCAGCGCCTCCTCACCCCCCAAGAAGGAGCAC
CACCACCATCACCACCACTCAGAGTCCCCAAAGGCCCCCGTGCCACTGCTCCCACCC
CTGCCCCCACCTCCACCTGAGCCCGAGAGCTCCGAGGACCCCACCAGCCCCCCTGAG
CCCCAGGACTTGAGCAGCAGCGTCTGCAAAGAGGAGAAGATGCCCAGAGGAGGCTC
ACTGGAGAGCGACGGCTGCCCCAAGGAGCCAGCTAAGACTCAGCCCGCGGTTGCCA
CCGCCGCCACGGCCGCAGAAAAGTACAAACACCGAGGGGAGGGAGAGCGCAAAGA
CATTGTTTCATCCTCCATGCCAAGGCCAAACAGAGAGGAGCCTGTGGACAGCCGGA
CGCCCGTGACCGAGAGAGTTAGCTGACTTTACACGGAGCGGATTGCAAAGCAAACC
AACAAGAATAAAGGCAGCTGTTGTCTCTTCTCCTTATGGGTAGGGCTCTGACAAAGC
TTCCCGATTAACTGAAATAAAAAATATTTTTTTTTCTTTCAGTAAACTTAGAGTTTCG
TGGCTTCAGGGTGGGAGTAGTTGGAGCATTGGGGATGTTTTTCTTACCGACAAGCAC
AGTCAGGTTGAAGACCTAACCAGGGCCAGAAGTAGCTTTGCACTTTTCTAAACTAGG
CTCCTTCAACAAGGCTTGCTGCAGATACTACTGACCAGACAAGCTGTTGACCAGGCA
CCTCCCCTCCCGCCCAAACCTTTCCCCCATGTGGTCGTTAGAGACAGAGCGACAGAG
CAGTTGAGAGGACACTCCCGTTTTCGGTGCCATCAGTGCCCCGTCTACAGCTCCCCC
AGCTCCCCCCACCTCCCCCACTCCCAACCACGTTGGGACAGGGAGGTGTGAGGCAG
GAGAGACAGTTGGATTCTTTAGAGAAGATGGATATGACCAGTGGCTATGGCCTGTG
CGATCCCACCCGTGGTGGCTCAAGTCTGGCCCCACACCAGCCCCAATCCAAAACTGG
CAAGGACGCTTCACAGGACAGGAAAGTGGCACCTGTCTGCTCCAGCTCTGGCATGG
CTAGGAGGGGGGAGTCCCTTGAACTACTGGGTGTAGACTGGCCTGAACCACAGGAG
AGGATGGCCCAGGGTGAGGTGGCATGGTCCATTCTCAAGGGACGTCCTCCAACGGG
TGGCGCTAGAGGCCATGGAGGCAGTAGGACAAGGTGCAGGCAGGCTGGCCTGGGGT
CAGGCCGGGCAGAGCACAGCGGGGTGAGAGGGATTCCTAATCACTCAGAGCAGTCT
GTGACTTAGTGGACAGGGGAGGGGGCAAAGGGGGAGGAGAAGAAAATGTTCTTCC
AGTTACTTTCCAATTCTCCTTTAGGGACAGCTTAGAATTATTTGCACTATTGAGTCTT
CATGTTCCC

Sequence S5. Consensus sequence of Edited Outcome 1 in R106W cells represented in Figure 5.

GCTCACAGTAAGGATGCCTAGATGGGGTTACCTGTTGTCAAGATAAGGAAACTGAA
GCACAGAATGCTGAGGTCATTTGCTGGGTTCATGTTTGGAAAGCGGCAAAGGATTTC
AGTGCAGGTTGGCTGGCTCCAAACCTGTGTGTGCTTTCCATGACACTGTACTGTGTG
CCTCATTGAGCCTCATTCTAGAAAACCAAAAACACACCCAAGGCCCGGCCTTCACA

AAGGAGACCCCTCCCCCATTTGGCTCCCTTTCCAGCAGTCGACGGCCTCTTGTCAGC
CATCGAGCCCAGAGTCCCTTGAAGTGCGACTCATGCTGGGGTGGTATGCTCAGGAGC
CGCAGTGTTTCCGCTCAGAGGAAAGGGCTCTGATTCTCCTGCAGTGCTAGGAGACTT
GTGGGTGGCCACAGTGCAGGTCAGGCACACCGGCCAGCACCACCCACAGCCCAAAT
TCCTAAAGAAATATTTGGGTCCCAGCTTGGCCCGAGTCTCTGTTGTCCTGGGGAAGG
ACATCAAGATCTGAGTGTATGATGGCCTGGGGCCTTGCATGTGGTGGGGGTCCAAGC
CTGCCTCTGCTCACTTGTTCTGCAGACTGGCATGTTCTCTGTGATACTTACATACTTG
TTTAACACTTCAGGGAAGAAAAGTCAGAAGACCAGGACCTCCAGGGCCTCAAGGAC
AAACCCCTCAAGTTTAAAAAGGTGAAGAAAGATAAGAAAGAAGAGAAAGAGGGCA
AGCATGAGCCCGTGCAGCCATCAGCCCACCACTCTGCTGAGCCCGCAGAGGCAGGC
AAAGCAGAGACATCAGAAGGGTCAGGCTCCGCCCCGGCTGTGCCGGAAGCTTCTGC
CTCCCCCAAACAGCGGCGCTCCATCATCCGTGACCGGGGACCCATGTATGATGACCC
CACCCTGCCTGAAGGCTGGACA**CGG**AAGCTTAAGCAAAGGAAATCTGGCCGCTCTG
CTGGGAAGTATGATGTGTATTTGATCAAGTAAGTAAGAGCAACTCCTATCTCTACAG
GGCAGGGAGGGCAGGGACAAGGATCCCTCATGGAGCAGGAAAATGTATGTGCCCA
GGGTGGGGTCGGGGGGAACATAAACAATGAACACTGAGACCAGGTGTGCTTGAAAT
GACCGTGTACAGAGGTCGCTGCCCTGAGTGGGAAGTTCTCAAGGTAGCAGGCCCTCT
ATCCTCTCCACACCTCAAGTCTTTATCTGGGGATGGAATAGCTGCGGAAGCAGAGGA
ACTTGCAGAGCCAGGGGTTCAGAGGGGTGAAGAAGCATGTTTCAGTTCTGCCTTTTA
AATGATCCCAAAAAGGTTAGCAGTTTTCAAATGACATTTGCAGACAGCCTCATTTAA
TTCCATGAGAAGGGTGAGCAAAGGATTATCTTGTTGAAACTGATTCCTGGAGAGACT
GAGCAC**ACTAGTGACTACAAAGACCATGACGG**

Sequence S6. Consensus sequence of Edited Outcome 2 in R106W cells represented in Figure 5.

GGGTTACCTGTTGTCAAGATAAGGAAACTGAAGCACAGAATGCTGAGGTCATTTGCT
GGGTTCATGTTTGGAAAGCGGCAAAGGATTTCAGTGCAGGTTGGCTGGCTCCAAACC
TGTGTGTGCTTTCCATGACACTGTACTGTGTGCCTCATTGAGCCTCATTCTAGAAAAC
CAAAAACACACCCAAGGCCCGGCCTTCACAAAGGAGACCCCTCCCCCATTTGGCTC
CCTTTCCAGCAGTCGACGGCCTCTTGTCAGCCATCGAGCCCAGAGTCCCTTGAAGTG
CGACTCATGCTGGGGTGGTATGCTCAGGAGCCGCAGTGTTTCCGCTCAGAGGAAAG
GGCTCTGATTCTCCTGCAGTGCTAGGAGACTTGTGGGTGGCCACAGTGCAGGTCAGG
CACACCGGCCAGCACCACCCACAGCCCAAATTCCTAAAGAAATATTTGGGTCCCAG
CTTGGCCCGAGTCTCTGTTGTCCTGGGGAAGGACATCAAGATCTGAGTGTATGATGG
CCTGGGGCCTTGCATGTGGTGGGGGTCCAAGCCTGCCTCTGCTCACTTGTTCTGCAG
ACTGGCATGTTCTCTGTGATACTTACATACTTGTTTAACACTTCAGGGAAGAAAAGT
CAGAAGACCAGGACCTCCAGGGCCTCAAGGACAAACCCCTCAAGTTTAAAAAGGTG
AAGAAAGATAAGAAAGAAGAGAAAGAGGGCAAGCATGAGCCCGTGCAGCCATCAG
CCCACCACTCTGCTGAGCCCGCAGAGGCAGGCAAAGCAGAGACATCAGAAGGGTCA
GGCTCCGCCCCGGCTGTGCCGGAAGCTTCTGCCTCCCCCAAACAGCGGCGCTCCATC
ATCCGTGACCGGGGACCCATGTATGATGACCCCACCCTGCCTGAAGGCTGGACA**TG**
GAAGCTTAAGCAAAGGAAATCTGGCCGCTCTGCTGGGAAGTATGATGTGTATTTGAT
CAAGTAAGTAAGAGCAACTCCTATCTCTACAGGGCAGGGAGGGCAGGGACAAGGAT
CCCTCATGGAGCAGGAAAATGTATGTGCCCAGGGTGGGGTCGGGGGGAACATAAAC

AATGAACACTGAGACCAGGTGTGCTTGAAATGACCGTGTACAGAGGTCGCTGCCCT
GAGTGGGAAGTTCTCAAGGTAGCAGGCCCTCTATCCTCTCCACACCTCAAGTCTTTA
TCTGGGGATGGAATAGCTGCGGAAGCAGAGGAACTTGCAGAGCCAGGGGTTCAGAG
GGGTGAAGAAGCATGTTTCAGTTCTGCCTTTTAAATGATCCCAAAAAGGTTAGCAGT
TTTCAAATGACATTTGCAGACAGCCTCATTTAATTCCATGAGAAGGGTGAGCAAAGG
ATTATCTTGTTGAAACTGATTCCTGGAGAGACTGAGCACA**CTAGTGACTACAAAGA
CCATGACGG**

Sequence S7. Consensus sequence of Edited Outcome 3 in R106W cells represented in Figure 5.

GCTCACAGTAAGGATGCCTAGATGGGGTTACCTGTTGTCAAGATAAGGAAACTGAA
GCACAGAATGCTGAGGTCATTTGCTGGGTTCATGTTTGGAAAGCGGCAAAGGATTTC
AGTGCAGGTTGGCTGGCTCCAAACCTGTGTGTGCTTTCCATGACACTGTACTGTGTG
CCTCATTGAGCCTCATTCTAGAAAACCAAAAACACACCCAAGGCCCGGCCTTCACA
AAGGAGACCCCTCCCCCATTTGGCTCCCTTTCCAGCAGTCGACGGCCTCTTGTCAGC
CATCGAGCCCAGAGTCCCTTGAAGTGCGACTCATGCTGGGGTGGTATGCTCAGGAGC
CGCAGTGTTTCCGCTCAGAGGAAAGGGCTCTGATTCTCCTGCAGTGCTAGGAGACTT
GTGGGTGGCCACAGTGCAGGTCAGGCACACCGGCCAGCACCACCCACAGCCCAAAT
TCCTAAAGAAATATTTGGGTCCCAGCTTGGCCCGAGTCTCTGTTGTCCTGG**GAGGTT
TCCCGGAACCTTTTGGAAAACC**GGAAGGACATCAAGATCTGAGTGTATGATGGCC
TGGGGCCTTGCATGTGGTGGGGGTCCAAGCCTGCCTCTGCTCACTTGTTCTGCAGAC
TGGCATGTTCTCTGTGATACTTACATACTTGTTTAACACTTCAGGGAAGAAAAGTCA
GAAGACCAGGACCTCCAGGGCCTCAAGGACAAACCCCTCAAGTTTAAAAAGGTGAA
GAAAGATAAGAAAGAAGAGAAAGAGGGCAAGCATGAGCCCGTGCAGCCATCAGCC
CACCACTCTGCTGAGCCCGCAGAGGCAGGCAAAGCAGAGACATCAGAAGGGTCAG
GCTCCGCCCCGGCTGTGCCGGAAGCTTCTGCCTCCCCCAAACAGCGGCGCTCCATCA
TCCGTGACCGGGGACCCATGTATGATGACCCCACCCTGCCTGAAGGCTGGACA**CGG**
AAGCTTAAGCAAAGGAAATCTGGCCGCTCTGCTGGGAAGTATGATGTGTATTTGATC
AAGTAAGTAAGAGCAACTCCTATCTCTACAGGGCAGGGAGGGCAGGGACAAGGATC
CCTCATGGAGCAGGAAAATGTATGTGCCCAGGGTGGGGTCGGGGGGAACATAAACA
ATGAACACTGAGACCAGGTGTGCTTGAAATGACCGTGTACAGAGGTCGCTGCCCTG
AGTGGGAAGTTCTCAAGGTAGCAGGCCCTCTATCCTCTCCACACCTCAAGTCTTTAT
CTGGGGATGGAATAGCTGCGGAAGCAGAGGAACTTGCAGAGCCAGGGGTTCAGAG
GGGTGAAGAAGCATGTTTCAGTTCTGCCTTTTAAATGATCCCAAAAAGGTTAGCAGT
TTTCAAATGACATTTGCAGACAGCCTCATTTAATTCCATGAGAAGGGTGAGCAAAGG
ATTATCTTGTTGAAACTGATTCCTGGAGAGACTGAGCACA**CTAGTGACTACAAAGA
CCATGACGG**

Sequence S8. Consensus sequence of Edited Outcome 4 in R106W cells represented in Figure 5.

GGGTTACCTGTTGTCAAGATAAGGAAACTGAAGCACAGAATGCTGAGGTCATTTGCT
GGGTTCATGTTTGGAAAGCGGCAAAGGATTTCAGTGCAGGTTGGCTGGCTCCAAACC
TGTGTGTGCTTTCCATGACACTGTACTGTGTGCCTCATTGAGCCTCATTCTAGAAAAC
CAAAAACACACCCAAGGCCCGGCCTTCACAAAGGAGACCCCTTCCCCATTTGGCTC
CCTTTCCAGCAGTCGACGGCCTCTTGTCAGCCATCGAGCCCAGAGTCCCTTGAAGTG

CGACTCATGCTGGGGTGGTATGCTCAGGAGCCGCAGTGTTTCCGCTCAGAGGAAAG
GGCTCTGATTCTCCTGCAGTGCTAGGAGACTTGTGGGTGGCCACAGTGCAGGTCAGG
CACACCGGCCAGCACCACCCACAGCCCAAATTCCTAAAGAAATATTTGGGTCCCAG
CTTGGCCCGAGTCTCTGTTGTCCTGG**GAGGTTTCCCGGAACCTTTTGGAAAACC**G
GAAGGACATCAAGATCTGAGTGTATGATGGCCTGGGGCCTTGCATGTGGTGGGGGT
CCAAGCCTGCCTCTGCTCACTTGTTCTGCAGACTGGCATGTTCTCTGTGATACTTACA
TACTTGTTTAACACTTCAGGGAAGAAAAGTCAGAAGACCAGGACCTCCAGGGCCTC
AAGGACAAACCCCTCAAGTTTAAAAAGGTGAAGAAAGATAAGAAAGAAGAGAAAG
AGGGCAAGCATGAGCCCGTGCAGCCATCAGCCCACCACTCTGCTGAGCCCGCAGAG
GCAGGCAAAGCAGAGACATCAGAAGGGTCAGGCTCCGCCCCGGCTGTGCCGGAAGC
TTCTGCCTCCCCCAAACAGCGGCGCTCCATCATCCGTGACCGGGGACCCATGTATGA
TGACCCCACCCTGCCTGAAGGCTGGACA**CGG**AAGCTTAAGCAAAGGAAATCTGGCC
GCTCTGCTGGGAAGTATGATGTGTATTTGATCAAGTAAGTAAGAGCAACTCCTATCT
CTACAGGGCAGGGAGGGCAGGGACAAGGATCCCTCATGGAGCAGGAAAATGTATGT
GCCCAGGGTGGGGTCGGGGGGAACATAAACAATGAACACTGAGACCAGGTGTGCTT
GAAATGACCGTGTACAGAGGTCGCTGCCCTGAGTGGGAAGTTCTCAAGGTAGCAGG
CCCTCTATCCTCTCCACACCTCAAGTCTTTATCTGGGGATGGAATAGCTGCGGAAGC
AGAGGAACTTGCAGAGC**C**AGGGGTTCAGAGGGGTGAAGAAGCATGTTTCAGTTCTG
CCTTTTAATGATCCCAAAAAGGTTAGCAGTTTTCAAATGACATTTGCAGACAGCCT
CATTTAATTCCATGAGAAGGGTGAGCAAAGGATTATCTTGTTGAAACTGATTCCTGG
AGAGACTGAGCAC**ACTAGTGACTACAAAGACCATGACGG**

Sequence S9. Consensus sequence of Edited Outcome B1 in R282X cells represented in Figure 6B.

GGGTTACCTGTTGTCAAGATAAGGAAACTGAAGCACAGAATGCTGAGGTCATTTGCT
GGGTTCATGTTTGGAAAGCGGCAAAGGATTTCAGTGCAGGTTGGCTGGCTCCAAACC
TGTGTGTGCTTTCCATGACACTGTACTGTGTGCCTCATTGAGCCTCATTCTAGAAAAC
CAAAAACACACCCAAGGCCCGGCCTTCACAAAGGAGACCCCT**T**CCCCATTTGGCTC
CCTTTCCAGCAGTCGACGGCCTCTTGTCAGCCATCGAGCCCAGAGTCCCTTGAAGTG
CGACTCATGCTGGGGTGGTATGCTCAGGAGCCGCAGTGTTTCCGCTCAGAGGAAAG
GGCTCTGATTCTCCTGCAGTGCTAGGAGACTTGTGGGTGGCCACAGTGCAGGTCAGG
CACACCGGCCAGCACCACCCACAGCCCAAATTCCTAAAGAAATATTTGGGTCCCAG
CTTGGCCCGAGTCTCTGTTGTCCTGG**GAGGTTTCCCGGAACCTTTTGGAAAACC**G
GAAGGACATCAAGATCTGAGTGTATGATGGCCTGGGGCCTTGCATGTGGTGGGGGT
CCAAGCCTGCCTCTGCTCACTTGTTCTGCAGACTGGCATGTTCTCTGTGATACTTACA
TACTTGTTTAACACTTCAGGGAAGAAAAGTCAGAAGACCAGGACCTCCAGGGCCTC
AAGGACAAACCCCTCAAGTTTAAAAAGGTGAAGAAAGATAAGAAAGAAGAGAAAG
AGGGCAAGCATGAGCCCGTGCAGCCATCAGCCCACCACTCTGCTGAGCCCGCAGAG
GCAGGCAAAGCAGAGACATCAGAAGGGTCAGGCTCCGCCCCGGCTGTGCCGGAAGC
TTCTGCCTCCCCCAAACAGCGGCGCTCCATCATCCGTGACCGGGGACCCATGTATGA
TGACCCCACCCTGCCTGAAGGCTGGACACGGAAGCTTAAGCAAAGGAAATCTGGCC
GCTCTGCTGGGAAGTATGATGTGTATTTGATCAAGTAAGTAAGAGCAACTCCTATCT
CTACAGGGCAGGGAGGGCAGGGACAAGGATCCCTCATGGAGCAGGAAAATGTATGT

GCCCAGGGTGGGGTCGGGGGGAACATAAACAATGAACACTGAGACCAGGTGTGCTT
GAAATGACCGTGTACAGAGGTCGCTGCCCTGAGTGGGAAGTTCTCAAGGTAGCAGG
CCCTCTATCCTCTCCACACCTCAAGTCTTTATCTGGGGATGGAATAGCTGCGGAAGC
AGAGGAACTTGCAGAGCCAGGGGTTCAGAGGGGTGAAGAAGCATGTTTCAGTTCTG
CCTTTTAAATGATCCCAAAAAGGTTAGCAGTTTTCAAATGACATTTGCAGACAGCCT
CATTTAATTCCATGAGAAGGGTGAGCAAAGGATTATCTTGTTGAAACTGATTCCTGG
AGAGACTGAGCACA**CTAGTGACTACAAAGACCATGACGG**

Sequence S10. Consensus sequence of Edited Outcome B2 in R282X cells represented in Figure 6B.

GGGTTACCTGTTGTCAAGATAAGGAAACTGAAGCACAGAATGCTGAGGTCATTTGCT
GGGTTCATGTTTGGAAAGCGGCAAAGGATTTCAGTGCAGGTTGGCTGGCTCCAAACC
TGTGTGTGCTTTCCATGACACTGTACTGTGTGCCTCATTGAGCCTCATTCTAGAAAAC
CAAAAACACACCCAAGGCCCGGCCTTCACAAAGGAGACCCCTCCCCCATTTGGCTC
CCTTTCCAGCAGTCGACGGCCTCTTGTCAGCCATCGAGCCCAGAGTCCCTTGAAGTG
CGACTCATGCTGGGGTGGTATGCTCAGGAGCCGCAGTGTTTCCGCTCAGAGGAAAG
GGCTCTGATTCTCCTGCAGTGCTAGGAGACTTGTGGGTGGCCACAGTGCAGGTCAGG
CACACCGGCCAGCACCACCCACAGCCCAAATTCCTAAAGAAATATTTGGGTCCCAG
CTTGGCCCGAGTCTCTGTTGTCCTGG**GAGGTTTCCCGGAACCTTTTGGAAAACC**G
GAAGGACATCAAGATCTGAGTGTATGATGGCCTGGGGCCTTGCATGTGGTGGGGGT
CCAAGCCTGCCTCTGCTCACTTGTTCTGCAGACTGGCATGTTCTCTGTGATACTTACA
TACTTGTTTAACACTTCAGGGAAGAAAAGTCAGAAGACCAGGACCTCCAGGGCCTC
AAGGACAAACCCCTCAAGTTTAAAAAGGTGAAGAAAGATAAGAAAGAAGAGAAAG
AGGGCAAGCATGAGCCCGTGCAGCCATCAGCCCACCACTCTGCTGAGCCCGCAGAG
GCAGGCAAAGCAGAGACATCAGAAGGGTCAGGCTCCGCCCCGGCTGTGCCGGAAGC
TTCTGCCTCCCCCAAACAGCGGCGCTCCATCATCCGTGACCGGGGACCCATGTATGA
TGACCCCACCCTGCCTGAAGGCTGGACACGGAAGCTTAAGCAAAGGAAATCTGGCC
GCTCTGCTGGGAAGTATGATGTGTATTTGATCAAGTAAGTAAGAGCAACTCCTATCT
CTACAGGGCAGGGAGGGCAGGGACAAGGATCCCTCATGGAGCAGGAAAATGTATGT
GCCCAGGGTGGGGTCGGGGGGAACATAAACAATGAACACTGAGACCAGGTGTGCTT
GAAATGACCGTGTACAGAGGTCGCTGCCCTGAGTGGGAAGTTCTCAAGGTAGCAGG
CCCTCTATCCTCTCCACACCTCAAGTCTTTATCTGGGGATGGAATAGCTGCGGAAGC
AGAGGAACTTGCAGAGCCAGGGGTTCAGAGGGGTGAAGAAGCATGTTTCAGTTCTG
CCTTTTAAATGATCCCAAAAAGGTTAGCAGTTTTCAAATGACATTTGCAGACAGCCT
CATTTAATTCCATGAGAAGGGTGAGCAAAGGATTATCTTGTTGAAACTGATTCCTGG
AGAGACTGAGCACA**CTAGTGACTACAAAGACCATGACGG**

Sequence S11. Consensus sequence of Edited Outcome B3 in R282X cells represented in Figure 6B.

GGGTTACCTGTTGTCAAGATAAGGAAACTGAAGCACAGAATGCTGAGGTCATTTGCT
GGGTTCATGTTTGGAAAGCGGCAAAGGATTTCAGTGCAGGTTGGCTGGCTCCAAACC
TGTGTGTGCTTTCCATGACACTGTACTGTGTGCCTCATTGAGCCTCATTCTAGAAAAC
CAAAAACACACCCAAGGCCCGGCCTTCACAAAGGAGACCCCTCCCCCATTTGGCTC

CCTTTCCAGCAGTCGACGGCCTCTTGTCAGCCATCGAGCCCAGAGTCCCTTGAAGTG
CGACTCATGCTGGGGTGGTATGCTCAGGAGCCGCAGTGTTTCCGCTCAGAGGAAAG
GGCTCTGATTCTCCTGCAGTGCTAGGAGACTTGTGGGTGGCCACAGTGCAGGTCAGG
CACACCGGCCAGCACCACCCACAGCCCAAATTCCTAAAGAAATATTTGGGTCCCAG
CTTGGCCCGAGTCTCTGTTGTCCTGGGGAAGGACATCAAGATCTGAGTGTATGATGG
CCTGGGGCCTTGCATGTGGTGGGGGTCCAAGCCTGCCTCTGCTCACTTGTTCTGCAG
ACTGGCATGTTCTCTGTGATACTTACATACTTGTTTAACACTTCAGGGAAGAAAAGT
CAGAAGACCAGGACCTCCAGGGCCTCAAGGACAAACCCCTCAAGTTTAAAAAGGTG
AAGAAAGATAAGAAAGAAGAGAAAGAGGGCAAGCATGAGCCCGTGCAGCCATCAG
CCCACCACTCTGCTGAGCCCGCAGAGGCAGGCAAAGCAGAGACATCAGAAGGGTCA
GGCTCCGCCCCGGCTGTGCCGGAAGCTTCTGCCTCCCCCAAACAGCGGCGCTCCATC
ATCCGTGACCGGGGACCCATGTATGATGACCCCACCCTGCCTGAAGGCTGGACACG
GAAGCTTAAGCAAAGGAAATCTGGCCGCTCTGCTGGGAAGTATGATGTGTATTTGAT
CAAGTAAGTAAGAGCAACTCCTATCTCTACAGGGCAGGGAGGGCAGGGACAAGGAT
CCCTCATGGAGCAGGAAAATGTATGTGCCCAGGGTGGGGTCGGGGGGAACATAAAC
AATGAACACTGAGACCAGGTGTGCTTGAAATGACCGTGTACAGAGGTCGCTGCCCT
GAGTGGGAAGTTCTCAAGGTAGCAGGCCCTCTATCCTCTCCACACCTCAAGTCTTTA
TCTGGGGATGGAATAGCTGCGGAAGCAGAGGAACTTGCAGAGCCAGGGGTTCAGAG
GGGTGAAGAAGCATGTTTCAGTTCTGCCTTTTAAATGATCCCAAAAAGGTTAGCAGT
TTTCAAATGACATTTGCAGACAGCCTCATTTAATTCCATGAGAAGGGTGAGCAAAGG
ATTATCTTGTTGAAACTGATTCCTGGAGAGACTGAGCACA**CTAGTGACTACAAAGA**
CCATGACGG

Sequence S12. Consensus sequence of Edited Outcome B4 in R282X cells represented in Figure 6B.

GGGTTACCTGTTGTCAAGATAAGGAAACTGAAGCACAGAATGCTGAGGTCATTTGCT
GGGTTCATGTTTGGAAAGCGGCAAAGGATTTCAGTGCAGGTTGGCTGGCTCCAAACC
TGTGTGTGCTTTCCATGACACTGTACTGTGTGCCTCATTGAGCCTCATTCTAGAAAAC
CAAAAACACACCCAAGGCCCGGCCTTCACAAAGGAGACCCCTCCCCCATTTGGCTC
CCTTTCCAGCAGTCGACGGCCTCTTGTCAGCCATCGAGCCCAGAGTCCCTTGAAGTG
CGACTCATGCTGGGGTGGTATGCTCAGGAGCCGCAGTGTTTCCGCTCAGAGGAAAG
GGCTCTGATTCTCCTGCAGTGCTAGGAGACTTGTGGGTGGCCACAGTGCAGGTCAGG
CACACCGGCCAGCACCACCCACAGCCCAAATTCCTAAAGAAATATTTGGGTCCCAG
CTTGGCCCGAGTCTCTGTTGTCCTGGGGAAGGACATCAAGATCTGAGTGTATGATGG
CCTGGGGCCTTGCATGTGGTGGGGGTCCAAGCCTGCCTCTGCTCACTTGTTCTGCAG
ACTGGCATGTTCTCTGTGATACTTACATACTTGTTTAACACTTCAGGGAAGAAAAGT
CAGAAGACCAGGACCTCCAGGGCCTCAAGGACAAACCCCTCAAGTTTAAAAAGGTG
AAGAAAGATAAGAAAGAAGAGAAAGAGGGCAAGCATGAGCCCGTGCAGCCATCAG
CCCACCACTCTGCTGAGCCCGCAGAGGCAGGCAAAGCAGAGACATCAGAAGGGTCA
GGCTCCGCCCCGGCTGTGCCGGAAGCTTCTGCCTCCCCCAAACAGCGGCGCTCCATC
ATCCGTGACCGGGGACCCATGTATGATGACCCCACCCTGCCTGAAGGCTGGACACG
GAAGCTTAAGCAAAGGAAATCTGGCCGCTCTGCTGGGAAGTATGATGTGTATTTGAT
CAAGTAAGTAAGAGCAACTCCTATCTCTACAGGGCAGGGAGGGCAGGGACAAGGAT

CCCTCATGGAGCAGGAAAATGTATGTGCCCAGGGTGGGGTCGGGGGGAACATAAAC
AATGAACACTGAGACCAGGTGTGCTTGAAATGACCGTGTACAGAGGTCGCTGCCCT
GAGTGGGAAGTTCTCAAGGTAGCAGGCCCTCTATCCTCTCCACACCTCAAGTCTTTA
TCTGGGGATGGAATAGCTGCGGAAGCAGAGGAACTTGCAGAGC**T**AGGGGTTCAGAG
GGGTGAAGAAGCATGTTTCAGTTCTGCCTTTTAAATGATCCCAAAAAGGTTAGCAGT
TTTCAAATGACATTTGCAGACAGCCTCATTTAATTCCATGAGAAGGGTGAGCAAAGG
ATTATCTTGTTGAAACTGATTCCTGGAGAGACTGAGCACA**CTAGTGACTACAAAGA
CCATGACGG**

Sequence S13. Consensus sequence of Edited Outcome C1 in R282X cells represented in Figure 6C.

CTAGTGACTACAAAGACCATGACGGTATCGATCGTACCTGAGTTCAAACTTGGGA
ATGTTCTAGATGGTGACTCAGGCCCAGGCACCAACCAGCAGAATGGGCCTCAGCCT
GACAACCCTTCTGTACCAGGCCTGACTCTTTGGTTGCTGAACTTTGGAGAGGCCTGG
GGGGGTCAGCGGCAGGCAGACGAGTGAGTGGCTTTGGTGACAGGTCCTCAGGGGCA
GCCAGGCAGTGTGACTCTCGTTCAATAGTAACGTTTGTCAGAGCGTTGTCACCACCA
TCCGCTCTGCCCTATCTCTGACATTGCTATGGAGAGCCTCTAATTGTTCCTTGTGTCT
TTCTGTTTGTCCCCACAGTCCCCAGGGAAAAGCCTTTCGCTCTAAAGTGGAGTTGAT
TGCGTACTTCGAAAAGGTAGGCGACACATCCCTGGACCCTAATGATTTTGACTTCAC
GGTAACTGGGAGAGGGAGCCCCTCCCGGCGAGAGCAGAAACCACCTAAGAAGCCC
AAATCTCCCAAAGCTCCAGGAACTGGCAGAGGCCGGGGACGCCCCAAAGGGAGCG
GCACCACGAGACCCAAGGCGGCCACGTCAGAGGGTGTGCAGGTGAAAAGGGTCCTG
GAGAAAAGTCCTGGGAAGCTCCTTGTCAAGATGCCTTTTCAAACTTCGCCAGGGGGC
AAGGCTGAGGGGGGTGGGGCCACCACATCCACCCAGGTCATGGTGATCAAACGCCC
CGGCAGGAAGCGAAAAGCTGAGGCCGACCCTCAGGCCATTCCCAAGAAACGGGGC
CGAAAGCCGGGGAGTGTGGTGGCAGCCGCTGCCGCCGAGGCCAAAAAGAAAGCCG
TGAAGGAGTCTTCTATCCGATCTGTGCAGGAGACCGTACTCCCCATCAAGAAGCGCA
AGACCCGGGAGACGGTCAGCATCGAGGTCAAGGAAGTGGTGAAGCCCCTGCTGGTG
TCCACCCTCGGTGAGAAGAGCGGGAAAGGACTGAAGACCTGTAAGAGCCCTGGGCG
GAAAAGCAAGGAGAGCAGCCCCAAGGGGCGCAGCAGCAGCGCCTCCTCACCCCCC
AAGAAGGAGCACCACCACCATCACCACCACTCAGAGTCCCCAAAGGCCCCCGTGCC
ACTGCTCCCACCCCTGCCCCCACCTCCACCTGAGCCCGAGAGCTCCGAGGACCCCAC
CAGCCCCCCTGAGCCCCAGGACTTGAGCAGC**AGT**GTCTGCAAAGAGGAGAAGATGC
CCAGAGGAGGCTCACTGGAGAGCGACGGCTGCCCCAAGGAGCCAGCTAAGACTCAG
CCCGCGGTTGCCACCGCCGCCACGGCCGCAGAAAAGTACAAACACCGAGGGGAGG
GAGAGCGCAAAGACATTGTTTCATCCTCCATGCCAAGGCCAAACAGAGAGGAGCCT
GTGGACAGCCGGACGCCCGTGACCGAGAGAGTTAGCTGACTTTACACGGAGCGGAT
TGCAAAGCAAACCAACAAGAATAAAGGCAGCTGTTGTCTCTTCTCCTTATGGGTAGG
GCTCTGACAAAGCTTCCCGATTAACTGAAATAAAAATATTTTTTTTTCTTTCAGTAA
ACTTAGAGTTTCGTGGCTTCAGGGTGGGAGTAGTTGGAGCATTGGGGATGTTTTTCT
TACCGACAAGCACAGTCAGGTTGAAGACCTAACCAGGGCCAGAAGTAGCTTTGCAC
TTTTCTAAACTAGGCTCCTTCAACAAGGCTTGCTGCAGATACTACTGACCAGACAAG
CTGTTGACCAGGCACCTCCCCTCCCGCCCAAACCTTTCCCCCATGTGGTCGTTAGAG

ACAGAGCGACAGAGCAGTTGAGAGGACACTCCCGTTTTCGGTGCCATCAGTGCCCC
GTCTACAGCTCCCCCAGCTCCCCCCACCTCCCCCACTCCCAACCACGTTGGGACAGG
GAGGTGTGAGGCAGGAGAGACAGTTGGATTCTTTAGAGAAGATGGATATGACCAGT
GGCTATGGCCTGTGCGATCCCACCCGTGGTGGCTCAAGTCTGGCCCCACACCAGCCC
CAATCCAAAACTGGCAAGGACGCTTCACAGGACAGGAAAGTGGCACCTGTCTGCTC
CAGCTCTGGCATGGCTAGGAGGGGGGAGTCCCTTGAACTACTGGGTGTAGACTGGC
CTGAACCACAGGAGAGGATGGCCCAGGGTGAGGTGGCATGGTCCATTCTCAAGGGA
CGTCCTCCAACGGGTGGCGCTAGAGGCCATGGAGGCAGTAGGACAAGGTGCAGGCA
GGCTGGCCTGGGGTCAGGCCGGGCAGAGCACAGCGGGGTGAGAGGGATTCCTAATC
ACTCAGAGCAGTCTGTGACTTAGTGGACAGGGGAGGGGGCAAAGGGGGAGGAGAA
GAAAATGTTCTTCCAGTTACTTTCCAATTCTCCTTTAGGGACAGCTTAGAATTATTTG
CACTATTGAGTCTTCATGTTCCCACTTCAAAACAAACAGATGCTCTGAGAGCAAACT
GGCTTGAATTGGTGACATTTAGTCCCTCAAGCCACCAGATGTGACAGTGTTGAGAAC
TACCTGGATTTGTATATATACCTGCGCTTGTTTTAAAGTGGGCTCAGCACATAGGGTT
CCCACGAAGCTCCGAAACTCTAAGTGTTTGCTGCAATTTTATAAGGACTTCCTGATT
GGTTTCTCTTCTCCCCTTCCATTTCTGCCTTTTGTTCATTTCATCCTTTCACTTCT

Sequence S14. Consensus sequence of Edited Outcome C2 in R282X cells represented in Figure 6C.

CTAGTGACTACAAAGACCATGACGGTATCGATCGTACCTGAGTTCAAACTTGGGA
ATGTTCTAGATGGTGACTCAGGCCCAGGCACCAACCAGCAGAATGGGCCTCAGCCT
GACAACCCTTCTGTACCAGGCCTGACTCTTTGGTTGCTGAACTTTGGAGAGGCCTGG
GGGGGTCAGCGGCAGGCAGACGAGTGAGTGGCTTTGGTGACAGGTCCTCAGGGGCA
GCCAGGCAGTGTGACTCTCGTTCAATAGTAACGTTTGTCAGAGCGTTGTCACCACCA
TCCGCTCTGCCCTATCTCTGACATTGCTATGGAGAGCCTCTAATTGTTCCTTGTGTCT
TTCTGTTTGTCCCCACAGTCCCCAGGGAAAAGCCTTTCGCTCTAAAGTGGAGTTGAT
TGCGTACTTCGAAAAGGTAGGCGACACATCCCTGGACCCTAATGATTTTGACTTCAC
GGTAACTGGGAGAGGGAGCCCCTCCCGGCGAGAGCAGAAACCACCTAAGAAGCCC
AAATCTCCCAAAGCTCCAGGAACTGGCAGAGGCCGGGGACGCCCCAAAGGGAGCG
GCACCACGAGACCCAAGGCGGCCACGTCAGAGGGTGTGCAGGTGAAAAGGGTCCTG
GAGAAAAGTCCTGGGAAGCTCCTTGTCAAGATGCCTTTTCAAACTTCGCCAGGGGGC
AAGGCTGAGGGGGGTGGGGCCACCACATCCACCCAGGTCATGGTGATCAAACGCCC
CGGCAGGAAGCGAAAAGCTGAGGCCGACCCTCAGGCCATTCCCAAGAAACGGGGC
TGAAAGCCGGGGAGTGTGGTGGCAGCCGCTGCCGCCGAGGCCAAAAAGAAAGCCG
TGAAGGAGTCTTCTATCCGATCTGTGCAGGAGACCGTACTCCCCATCAAGAAGCGCA
AGACCCGGGAGACGGTCAGCATCGAGGTCAAGGAAGTGGTGAAGCCCCTGCTGGTG
TCCACCCTCGGTGAGAAGAGCGGGAAAGGACTGAAGACCTGTAAGAGCCCTGGGCG
GAAAAGCAAGGAGAGCAGCCCCAAGGGGCGCAGCAGCAGCGCCTCCTCACCCCCC
AAGAAGGAGCACCACCACCATCACCACCACTCAGAGTCCCCAAAGGCCCCCGTGCC
ACTGCTCCCACCCCTGCCCCCACCTCCACCTGAGCCCGAGAGCTCCGAGGACCCCAC
CAGCCCCCCTGAGCCCCAGGACTTGAGCAGC**AGT**GTCTGCAAAGAGGAGAAGATGC
CCAGAGGAGGCTCACTGGAGAGCGACGGCTGCCCCAAGGAGCCAGCTAAGACTCAG
CCCGCGGTTGCCACCGCCGCCACGGCCGCAGAAAAGTACAAACACCGAGGGGAGG

GAGAGCGCAAAGACATTGTTTCATCCTCCATGCCAAGGCCAAACAGAGAGGAGCCT
GTGGACAGCCGGACGCCCGTGACCGAGAGAGTTAGCTGACTTTACACGGAGCGGAT
TGCAAAGCAAACCAACAAGAATAAAGGCAGCTGTTGTCTCTTCTCCTTATGGGTAGG
GCTCTGACAAAGCTTCCCGATTAACTGAAATAAAAAATATTTTTTTTTCTTTCAGTAA
ACTTAGAGTTTCGTGGCTTCAGGGTGGGAGTAGTTGGAGCATTGGGGATGTTTTTCT
TACCGACAAGCACAGTCAGGTTGAAGACCTAACCAGGGCCAGAAGTAGCTTTGCAC
TTTTCTAAACTAGGCTCCTTCAACAAGGCTTGCTGCAGATACTACTGACCAGACAAG
CTGTTGACCAGGCACCTCCCCTCCCGCCCAAACCTTTCCCCCATGTGGTCGTTAGAG
ACAGAGCGACAGAGCAGTTGAGAGGACACTCCCGTTTTCGGTGCCATCAGTGCCCC
GTCTACAGCTCCCCCAGCTCCCCCCACCTCCCCCACTCCCAACCACGTTGGGACAGG
GAGGTGTGAGGCAGGAGAGACAGTTGGATTCTTTAGAGAAGATGGATATGACCAGT
GGCTATGGCCTGTGCGATCCCACCCGTGGTGGCTCAAGTCTGGCCCCACACCAGCCC
CAATCCAAAACTGGCAAGGACGCTTCACAGGACAGGAAAGTGGCACCTGTCTGCTC
CAGCTCTGGCATGGCTAGGAGGGGGGAGTCCCTTGAACTACTGGGTGTAGACTGGC
CTGAACCACAGGAGAGGATGGCCCAGGGTGAGGTGGCATGGTCCATTCTCAAGGGA
CGTCCTCCAACGGGTGGCGCTAGAGGCCATGGAGGCAGTAGGACAAGGTGCAGGCA
GGCTGGCCTGGGGTCAGGCCGGGCAGAGCACAGCGGGGTGAGAGGGATTCCTAATC
ACTCAGAGCAGTCTGTGACTTAGTGGACAGGGGAGGGGGCAAAGGGGGAGGAGAA
GAAAATGTTCTTCCAGTTACTTTCCAATTCTCCTTTAGGGACAGCTTAGAATTATTTG
CACTATTGAGTCTTCATGTTCCCACTTCAAAACAAACAGATGCTCTGAGAGCAAACT
GGCTTGAATTGGTGACATTTAGTCCCTCAAGCCACCAGATGTGACAGTGTTGAGAAC
TACCTGGATTTGTATATATACCTGCGCTTGTTTTAAAGTGGGCTCAGCACATAGGGTT
CCCACGAAGCTCCGAAACTCTAAGTGTTTGCTGCAATTTTATAAGGACTTCCTGATT
GGTTTCTCTTCTCCCCTTCCATTTCTGCCTTTTGTTCATTTCATCCTTTCACTTCT

Supplementary Figure Legends

Figure S1. 5' Sequence analysis of edited MECP2 gene in S134C cells. A. Specific PCR product (1.4 kb) obtained with the edited hemizygous male S134C Rett syndrome cells with 5' TI assay (M: Marker; UT: Untransduced S134C cells; T: Transduced S134C cells). **B.** Sanger Sequence analysis of the edited MECP2 gene in hemizygous male S134C Rett syndrome cells. Shown are the chromatograms of 5' genome-vector junction, SNP in Intron 2, Linker 1 sequence and Linker 2 sequence, representing the 2 editing outcomes (B1 and B2) observed in S134C cells with the 5' TI assay. The primers used for 5' TI analyses are depicted as thick red arrows.

Figure S2. 3' Sequence analysis of edited MECP2 gene in S134C cells. A. Specific PCR product (2.7 kb) obtained with the edited hemizygous male S134C Rett syndrome cells with 3' TI assay (M: Marker; UT: Untransduced S134C cells; T: Transduced S134C cells). **B.** Sequence analysis of the edited MECP2 gene in hemizygous male S134C Rett syndrome cells. Shown are the chromatograms of Linker 2 sequence, codon encoding WT S or mutant C at position 134, Exon 4-3' UTR junction sequence and 3' vector-genome junction sequence, representing the 2 editing outcomes (C1 and C2) observed in S134C cells with the 3' TI assay. The primers used for 3' TI analyses are depicted as thick red arrows.

Figure S3. 5' Sequence analysis of edited MECP2 gene in R106W cells A. Specific PCR product (1.5 kb) obtained with the edited heterozygous female R106W Rett syndrome cells with 5' TI assay (M: Marker; UT: Untransduced R106W cells; T: Transduced R106W cells). **B.** Sequence analysis of the edited MECP2 gene in R106W Rett syndrome cells. Shown are the chromatograms of 5' genome-vector junction, SNP in Intron 2, Linker 1 sequence, codon encoding WT R or mutant W at position 106, SNP in Intron 3 and Linker 2 sequence, representing the 4 editing outcomes (1, 2, 3 and 4) observed in R106W cells with the 5' TI assay. The primers used for 5' TI analyses are depicted as thick red arrows.

Figure S4. 5' Sequence analysis of edited MECP2 gene in R282X cells. A. Specific PCR product (1.4 kb) obtained with the edited heterozygous female R282X Rett syndrome cells with 5' TI assay (M: Marker; UT: Untransduced R282X cells; T: Transduced R282X cells). **B.** Sequence analysis of the edited MECP2 gene in R282X Rett syndrome cells. Shown are the chromatograms of 5' genome-vector junction, SNP in Intron 2, Linker 1 sequence, SNP in Intron 3 and Linker 2 sequence, representing the 4 editing outcomes (B1, B2, B3 and B4) observed in R282X cells with the 5' TI assay. The primers used for 5' TI analyses are depicted as thick red arrows.

Figure S5. 3' Sequence analysis of edited MECP2 gene in R282X cells. A. Specific PCR product (2.7 kb) obtained with the edited heterozygous female R282X Rett syndrome cells with 3' TI assay (M: Marker; UT: Untransduced R282X cells; T: Transduced R282X cells). **B.** Sequence analysis of the edited MECP2 gene in R282X Rett syndrome cells. Shown are the chromatograms of Linker 2 sequence, codon encoding WT R or mutant X at position 282, SNP encoding for S at position 423, Exon 4- 3' UTR junction sequence and 3' vector-genome junction sequence, representing the 2 editing outcomes (C1 and C2) observed in R282X cells with the 3' TI assay. The primers used for 3' TI analyses are depicted as thick red arrows.

Figure S6. Restoration of MeCP2 expression in edited male hemizygous GM21921 (r.378_384del) fibroblasts. Mean MeCP2 fluorescence intensity (MFI) in untransduced (Untd) GM21921 cells and AAVHSC7-226 edited GM21921 cells. Data shows the average of MFI from 2 separate experiments. Bars represent the standard deviation.
